# Amiloride sensitizes prostate cancer cells to the reversible tyrosine kinase inhibitor lapatinib by modulating Erbb3 subcellular localization

**DOI:** 10.1007/s00018-024-05540-5

**Published:** 2024-12-27

**Authors:** Maitreyee K. Jathal, Maria Mudryj, Marc A. Dall’Era, Paramita M. Ghosh

**Affiliations:** 1 Research Service, VA Northern California Health Care System, Mather, CA USA; 2 Department of Medical Microbiology and Immunology, University of California Davis, Davis, CA USA; 3 Department of Urologic Surgery, University of California Davis, 4860 Y Street, Suite 3500, Sacramento, CA 95817 USA; 4 Department of Biochemistry and Molecular Medicine, University of California Davis, Sacramento, CA USA

**Keywords:** Amiloride, Lapatinib, ErbB3, Subcellular localization, Heregulin-1β, Androgen receptor, Prostate cancer

## Abstract

Neoadjuvant therapy (NAT) has been studied in clinically localized prostate cancer (PCa) to improve the outcomes from radical prostatectomy (RP) by ‘debulking’ of high-risk PCa; however, using androgen deprivation therapy (ADT) at this point risks castration resistant PCa (CRPC) clonal proliferation. Our goal is to identify alternative NAT that reduce hormone sensitive PCa (HSPC) without affecting androgen receptor (AR) transcriptional activity. PCa is associated with increased expression and activation of the epidermal growth factor receptor (EGFR) family, including HER2 and ErbB3. The FDA-approved HER2 inhibitor lapatinib has been tested in PCa but was ineffective due to continued activation of ErbB3. We now demonstrate that this is due to ErbB3 being localized to the nucleus in HSPC and thus protected from lapatinib which affect membrane localized HER2/ErbB3 dimers. Here, we show that the well-established, well-tolerated potassium-sparing diuretic amiloride hydrochloride dose dependently prevented ErbB3 nuclear localization via formation of plasma membrane localized HER2/ErbB3 dimers. This in turn allowed lapatinib inactivation of these dimers via inhibition of its target HER2, which dephosphorylated ERK1/2 and inhibited survival. Amiloride combined with lapatinib significantly increased apoptosis at relatively low doses of both drugs but did not affect AR transcriptional activity. Thus, our data indicate that a combination of amiloride and lapatinib could target HSPC tumors without problems associated with using ADT as NAT in HSPC.

**Supplementary Information:**

The online version contains supplementary material available at 10.1007/s00018-024-05540-5.

## Introduction

Localized prostate cancer (PCa) is often treated initially with radical prostatectomy (RP) or radiation therapy (RT), with an overall success rate of up to 90% alone [1]. High grade and more advanced tumors, however, may require multi-modal therapy as 25–33% of men initially treated with these therapies eventually experience biochemical recurrence [2]. While positive surgical margins at the time of RP are a risk factor for disease recurrence, many cancers are likely also micro-metastatic at the time of detection [3]. Neoadjuvant therapy (NAT) has been investigated as a method to debulk tumors and treat early systemic disease prior to RP [4]. NAT is also known to reduce post-operative residual local disease and micrometastases [5]. This eliminates the need for salvage therapy after RP such as radiation or long term hormonal therapy [6].

PCa is initially dependent on the androgen receptor (AR), a nuclear hormone transcription factor activated by binding to androgens such as testosterone (T) and dihydrotestosterone (DHT) [7]. With the advent of safe and reversible forms of androgen deprivation therapy (ADT) with or without antiandrogens, neoadjuvant ADT (NADT) is of significant interest [8]. However, following prolonged exposure, many patients develop resistance to ADT, resulting in castration resistant PCa (CRPC) [7], which demonstrates the limitations of AR-based PCa therapy. Similarly, NADT also runs the risk of androgen-independent clonal proliferation with prolonged treatment [9]. In addition, there may be impairments in quality of life due to profound side effects such as fatigue, loss of libido, hot flashes, loss of muscle mass, and weight gain [10]. To overcome these problems, other therapies are being studied in the neoadjuvant setting, including PARP inhibitors, while tyrosine kinase inhibitors have been tried in preclinical and early clinical studies [11].

We and others have demonstrated that PCa is often associated with an increase in the activation of receptor tyrosine kinases (RTK) of the epidermal growth factor receptor (EGFR) family, including EGFR, HER2/ErbB2 and HER3/ErbB3 (HER4/ErbB4 is rarely expressed in PCa) [12, 13]. HER2-4 initially referred to the protein form whereas ErbB referred to the corresponding gene; however, investigators have increasingly referred to the protein form as ErbB as well. Here, to prevent confusion between HER2 and HER3, we will refer to the latter as ErbB3. Our initial results showed that dual inhibition of EGFR and HER2 suppressed ErbB3 and sensitized PCa tumors to ADT [14]. Members of the EGFR family are activated by ligand binding. EGFR has a number of ligands– including epidermal growth factor (EGF), while ErbB3 is activated by heregulins 1 and 2 (HRG1, HRG2) [15]. Following ligand binding, these receptors undergo configurational alterations that allow heterodimerization with other members of the family. HER2 is known to be an orphan receptor that is constitutively active and hence does not require ligand binding for heterodimerization [15]. For complete activation, all receptors undergo autophosphorylation at various tyrosine residues that bind downstream targets [15].

Many clinical trials have been conducted to evaluate the effects of FDA approved tyrosine kinase inhibitors (TKI) targeting EGFR in PCa– including cetuximab, gefitinib and erlotinib [16–18]. While a few trials showed moderate results in a subpopulation of PCa patients (e.g. erlotinib had moderate single-agent activity in chemotherapy-naïve CRPC, while cetuximab had some activity in those overexpressing EGFR and showing consistent expression of the tumor suppressor PTEN) [18, 19], the majority of these trials failed to demonstrate efficacy. Of TKIs targeting HER2, pertuzumab was somewhat effective, while trastuzumab was ineffective as a single agent or in combination with the chemotherapeutic agent docetaxel [20]. In contrast, the dual EGFR/HER2 inhibitor lapatinib showed single agent activity in a small subset of patients [21]. The overall goal of the present project is to expand the efficacy of lapatinib in additional PCa patients in order to be used as a plausible NAT. The advantage of the FDA approved lapatinib is its low toxicity and high tolerability [22]. We previously showed that the pan-ErbB inhibitor dacomitinib was superior to lapatinib in preventing PCa progression [23]; however, dacomitinib has greater side effects; hence, we investigated whether lapatinib efficacy could be improved with another low toxic drug.

We recently showed that ErbB3 is localized to the membrane/cytoplasm in benign prostate but shows nuclear translocation in malignant prostate [24]. ADT increased ErbB3 cytoplasmic localization, whereas ErbB3 binding to its ligand heregulin-1β (HRG) induced ErbB3 nuclear localization [24]. PCa-specific nuclear expression of ErbB3 has long been recognized [25]. While an 80 kDa nuclear variant of ErbB3 has been identified [26], full-length 185 kDa ErbB3 also translocates to the nucleus in PCa [27]. We and others showed that increased nuclear localization of ErbB3 is associated with PCa progression [24, 25, 28].

Both EGFR and ErbB3 have been shown to undergo nuclear translocation through endocytosis [29, 30]. It is thought that internalization of ErbB3 initiates its entry into the nucleus where it interacts with the transcription complex and plays a role in transcriptional regulation, enabling PCa progression [29, 30]. Amiloride hydrochloride, a guanidinium-containing pyrazine derivative, has been shown to block ErbB3 nuclear translocation [30]. Amiloride is used as a potassium-sparing diuretic to treat hypertension by inhibiting the plasma membrane localized sodium-hydrogen exchanger protein 1 (NHE1), that plays a central role in intracellular pH and cell volume homeostasis [31, 32]. NHE1 activity is required to promote actin polymerization during macropinocytosis, explaining amiloride’s ability to antagonize this process [33]. Amiloride can also prevent hypokalemia by inhibiting the epithelial sodium channel (ENaC) [34]. Hypokalemia (low potassium levels) is a common side effect of lapatinib [35], caused by excessive phosphorylation of hERG potassium channels [36], which may result in cardiac toxicity [35]. while hyperkalemia is a side effect of amiloride [34, 37], and hence can counter this effect.

Amiloride may impede PCa tumor growth by inhibiting the urokinase-type plasminogen activator (uPA), a serine protease mediator of cell migration, invasion and metastasis and well‐known marker of poor prognosis in various human cancers, including PCa [38, 39]. Studies have revealed the anti-tumor properties of amiloride [32]. Amiloride decreases the invasiveness of highly-aggressive VCaP and ERG-transformed normal prostate cells [40] and decreases the sizes of PCa xenografts in immunodeficient rodents [41, 42]. In addition, amiloride inhibited the internalization of ErbB3 [30] and enhanced the effectiveness of the EGFR inhibitor erlotinib in pancreatic cancer [43] and the BCR-ABL inhibitor imatinib in leukemia [44].

In this paper, we show that EGFR and HER2 were mainly plasma membrane located in PCa cells while ErbB3 localized on the plasma membrane, the cytoplasm or the nucleus; in the plasma membrane, HER2 was activated by dimerization with ErbB3 and enhanced downstream signaling and cell proliferation. However, the HER2 inhibitor lapatinib was able to inhibit HER2/ErbB3 dimers and reduce its proliferative effects mainly when it was plasma membrane localized. Here we show that amiloride promoted ErbB3 translocation from the nucleus to the cytoplasm and the cytoplasm to the plasma membrane; as a result, amiloride enhanced HER2/ErbB3 dimerization that could then be inhibited by lapatinib. Taken together, our data suggest that amiloride enhances lapatinib activity by limiting ErbB3 to the plasma membrane and/or cytoplasm and enabling HER2/ErbB3 dimerization, which allows lapatinib to inhibit the dimer and prevent downstream activation of Akt and ERK. Thus, this combination of lapatinib and amiloride will be considered a means of drug ‘re-purposing’, that is effective in HSPC, and hence may in the future be used in NAT for the treatment of PCa patients.

## Materials and methods

### Cell culture and materials

Human prostatic carcinoma epithelial cell lines LNCaP, PC-346 C, C4-2 and CWR22-Rv1 (ATCC, Manassas, VA) were cultured in RPMI 1640 medium with 10% fetal bovine serum (Gemini Biologicals, West Sacramento, CA) and 1% antibiotic-antimycotic solutions (Gibco/Thermo Fisher Scientific, Waltham, MA). Lapatinib was purchased from Selleck Chemicals (Houston, TX). Amiloride hydrochloride was purchased from Amresco (VWR International, Radnor, PA). Heregulin-1β (HRG) was purchased from PeproTech (Rocky Hill, NJ). Rabbit monoclonal antibodies for EGFR (CS-2232), HER2 (CS-2165), ErbB3 (CS-12708) and Lamin A/C (CS-2032) were from Cell Signaling Technology (Beverly, MA). Mouse monoclonal antibody towards N-terminal ErbB3, OP-119 was purchased from Calbiochem/Millipore (San Diego, CA). ΜltraCruz Hard-set Mounting Medium was purchased from Santa Cruz BioTech (Dallas, TX).

### Subcellular fractionation

Cells were lysed for 15 m at room temperature in 500–900 µl of cytoplasmic lysis buffer A (10mM HEPES pH 7.9, 10mM KCl, 0.1mM EDTA, 0.4% IGEPAL) with standard protease and phosphatase inhibitors. The resulting suspension was centrifuged at 16,000 g for 5 m at 4 C and the supernatant was transferred to a clean 1.5 ml tube and stored at -20 C until further use. The pellet was washed thrice with 200–500 µl 1X phosphate-buffered saline (Gibco, Thermo Scientific, Waltham, MA) (5 m, 16000 g, 4 C) and reconstituted in ~ 150–300 µl of 1X sodiµM dodecyl sµlphate (SDS) Sample Buffer (10 g SDS, 4mls 100% glycerol, 40mls 1 M Tris-HCl pH 8.8, made upto 100mls with doubly distilled water). The pellet was heated at 90 C until it had completely dissolved, cooled to room temperature and stored at -20 C until further use.

### Immunofluorescence

LNCaP, C4-2, PC-346 C or 22Rv1 cells were seeded at 10,000 cells per coverslip and were incubated for 24 h in FBS medium in a 37^0^C CO_2_ incubator. Cells were treated with vehicle or drug for 72 h, rinsed with PBST (Phosphate Buffered Saline with 0.05% Tween-20) and fixed with ice-cold methanol for 10 min on ice. They were washed three times with PBST and then blocked with 5% BSA for 1 h at room temperature. Primary antibody was diluted 1:100 in 1% BSA and applied to the cells and incubated at 4^o^C overnight in a Humidity chamber. Cells were washed three times with PBST and the rhodamine or FITC-conjugated anti-rabbit or anti-mouse secondary antibodies (Life Technologies, Carlsbad, CA) were diluted 1:500 in PBST and incubated for 1 h at room temperature in the dark. After washing thrice with cold PBST, coverslips were inverted and mounted onto uncharged glass slides with UltraCruz Hardset Mounting Medium plus DAPI (SantaCruz BioTech, Dallas, TX). Slides were imaged at room temperature with a universal insert on an ECHO Revolve microscope (BICO, San Diego, CA) with a 5 MP CMOS monochrome camera using 40X magnification with filters for DAPI (EX:380/30, EM:450/50), FITC (EX:470/70, EM:525/50) and TRITC/Rhodamine (EX:530/40, EM:605/70). Captured images were transferred and processed using standard protocols to generate merged overlays with the publicly available ImageJ software (https://imagej.net/ij/).

### 3-[4,5-Dimethylthiazol-2yl]-2,5-diphenyl-tetrazolium bromide assay

Cells were cultured in 24-well plates and treated as indicated. Following treatment, each well was incubated with 25 µl of 5 mg/ml 3-[4,5-dimethylthiazol-2yl]-2,5-diphenyl-tetrazoliuM bromide (MTT; Sigma–Aldrich, St. Louis, MO) for 1 h in a 5% CO2 incubator at 37 °C, which converted the reactants to formazan in actively dividing cells. Proliferation rates were estimated by colorimetric assay reading formazan intensity in a plate reader at 562 nm.

### Western blotting

Proteins were quantitated by BCA assay (Pierce, Rockford, IL, USA) and fractionated on 29:1 acrylamide-bis SDS–PAGE. Electrophoresis was performed at 150 V for 2 h using mini vertical electrophoresis cells (Mini-PROTEAN 3 Electrophoresis Cell, Bio-Rad). The gels were electroblotted for 2 h at 200 mA using Mini Trans-Blot Electrophoretic Transfer Cell (Bio-Rad) onto 0.2 µM polyvinylidene difluoride membrane (Osmonics, Westborough, MA, USA). The blots were stained overnight with primary antibodies at 4 °C and detected by enhanced chemiluminescence (Thermo Fisher, Waltham, MA) following incubation with a peroxidase-labeled secondary antibody (donkey anti-mouse IgG or goat anti-rabbit IgG, Fc specific, Jackson ImmunoResearch, West Grove, PA, USA).

### Flow cytometry for apoptosis

Cells were grown in 35 mm plates at 250,000 cells per well in triplicate and treated with lapatinib, amiloride or the combination for 72 h. Cells were conjugated to Annexin V and propidium iodide per the manufacturer’s instructions (FITC Annexin V/Dead Cell Apoptosis Kit; Cat. No. V13242; Thermo Fisher Scientific, Inc.). Flow cytometry was then performed on FACSAria (Becton Dickinson Immunocytometry Systems, San Jose, CA, USA). Cells were illuminated with 200 mW of 488 nm light or 635 nm light. Fluorescence was detected through a 630/22 nm (for PI) or 661/16 nm (for Annexin V-Alexa Fluor 647) band-pass filter. Frequency histograms were collected from 20,000 events and analyzed in FlowJo software version 10.8.1 (TreeStar, FlowJo LLC., Ashland, OR, USA).

### qPCR

Total cellular RNA was prepared utilizing the Qiagen RNeasy kit (Redwood City, CA) based on the manufacturer’s protocol. cDNA was synthesized from 1 mg RNA using the iScript cDNA Synthesis Kit from BioRad (Hercules, CA) as per the manufacturer’s protocol. Real-time PCR was run using TaqMan Gene Expression Master Mix (Applied Biosystems, Grand Island, NY) according to manufacturer’s recommendations. B-Actin was used as the endogenous expression standard. Data were collected on an Applied Biosystems 7500 Fast machine and analyzed using the relative standard curve method.

### AR transcriptional activity

For reporter gene assays to investigate AR transcriptional activity, cells were plated in 6-well-plates and allowed to attach overnight before being transfected with 500 ng of commercially available pGL4-Luc or pGL4-hPSA-Luc constructs (Promega). For cotransfections, 2.5 µl of the appropriate siRNA or 500 ng of the appropriate DNA construct were used. After removal of the construct–lipid complexes, cells were treated with the appropriate medium and ligand conditions and allowed to incubate at 37 °C/5% CO2 for 48 h before being collected, lysed, and analyzed for AR transcriptional activity using a commercially available kit as per manufacturer’s instructions (E4550, Promega). Experiments were performed in triplicate.

### Plasmid construction

To create mutant ErbB3, the three desired mutations (Y1222A, Y1289A, and Y1325A) were introduced into the ErbB3 sequence (using the full-length WT ErbB3 plasmid kindly provided by Dr Kermit L. Carraway III, University of California Davis). The mutated ErbB3 sequence was synthesized with Not1 and Pml1 restriction sites at either end. The construct thus obtained was cut with the Not1 and Pml1 restriction enzymes and ligated into pcDNA3.1 (Invitrogen, Thermo Fisher Scientific). The ligation mix was transformed into Top10 competent cells (Invitrogen). The colonies were screened for the insert, inoculated for plasmid isolation, and were commercially sequence-verified.

### Statistical analysis

For all immunoblots, data were normalized first to loading controls (housekeeping genes such as tubulin, GAPDH and HSP90) and then to DMSO. For all other experiments data were normalized to DMSO. All statistical analyses were conducted by GraphPad Prism Version 10.2.0. p-values were calculated by unpaired t-test with Welch’s correction, while variances were compared by F-test. Unless otherwise indicated, *0.01 < *p* < 0.05, **0.001 < *p* < 0.01 and ***0.0001 < *p* < 0.001.

Additional details for plasmid construction, siRNA and transfections have been described in detail previously by us [23, 45].

## Results

### Amiloride promoted ErbB3 translocation from the nucleus to the cytoplasm and the plasma membrane

**Fig. 1:**
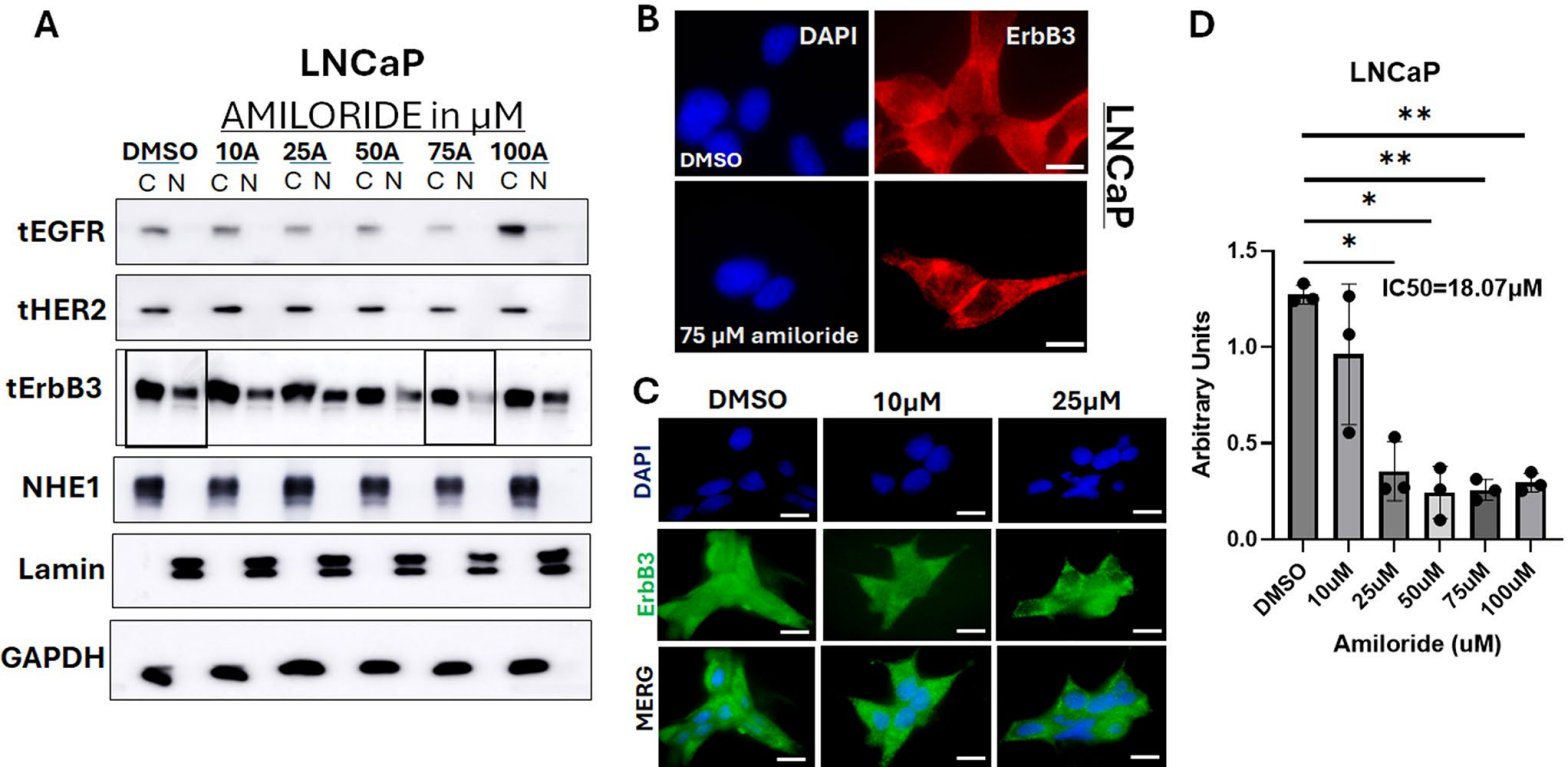
Amiloride promoted ErbB3 translocation from the nucleus to the cytoplasm and the plasma membrane in HSPC cells (**A**) Hormone-sensitive LNCaP cells were treated with varying concentrations of amiloride for 72 h before being lysed, fractionated and analyzed by immunoblot. (**B**) Immunofluorescence microscopy (IF) in LNCaP cells treated with DMSO or 75 µM or and probed with IF specific C-terminal ErbB3 antibodies or (**C**) 10µM or 25µM amiloride and probed with IF specific N-terminal ErbB3 antibodies for 72 h (scale bars = 30 μm). Note that vehicle treated LNCaP cells expressed nuclear ErbB3 (red) whereas amiloride-treated cells had significantly decreased ErbB3 expression in the nucleus (hollowed out). Location of nuclei are identified by blue DAPI staining. Plasma membrane localization of ErbB3 at cell-cell junction was also noted in amiloride-treated but not in vehicle treated cells. Note that both N- and C-terminal ErbB3 antibodies demonstrate lighter nuclear staining in amiloride-treated cells. (**D**) Cells were subjected to viability assays using the stated concentrations of amiloride. p-values are calculated with respect to DMSO

We compared the effects of increasing doses of amiloride for 72 h in hormone-sensitive LNCaP cells and their castration-resistant derivative C4-2 cells, as well as in an unrelated hormone-insensitive cell line CWR22Rv1 (denoted henceforth as 22Rv1). We have previously shown that all three cell lines express abundant ErbB3, HER2 and EGFR protein and mRNA (but not ErbB4) [23, 24]. Amiloride has been used at concentrations up to 1 mM in numerous cancer cell lines, including PCa [30, 46], but we tested the response of ErbB family kinases at amiloride concentrations only up to 100 µM. In LNCaP cells, baseline EGFR and HER2 were mostly cytoplasmic, with EGFR levels decreasing steadily until 75 µM, and then abruptly increasing at 100 µM (Fig. 1A). ErbB3, in contrast, displayed both cytoplasmic and nuclear localization at baseline; however, with increasing amiloride the ratio of cytoplasmic to nuclear ErbB3 steadily increased, until at 75µM amiloride it was significantly cytoplasmic. This result was validated using immunofluorescence microscopy which showed nuclear ErbB3 in vehicle-treated cells but not in 75 µM amiloride-treated cells (Fig. 1B). Surprisingly, at 100 µM amiloride, the levels of nuclear ErbB3 appeared to be restored, and the levels of cytoplasmic EGFR appeared to be increased. Lower doses of amiloride were examined as well and demonstrated smaller changes in localization in LNCaP cells (Fig. 1C); however, it is obvious that at 25 µM, ErbB3 translocated from a nuclear location to a cytoplasmic location, while at 10 µM, the level of nuclear ErbB3 is somewhat reduced. In parallel, amiloride also caused a dose dependent inhibition in cell growth, as indicated by MTT assay, with an IC_50_ = 18.07 µM (Fig. 1D). Significant suppression of cell growth was observed at 25 µM (*p* = 0.0236), and higher doses, suggesting correlation between loss of cell viability vs. loss of nuclear ErbB3 localization.

**Fig. 2:**
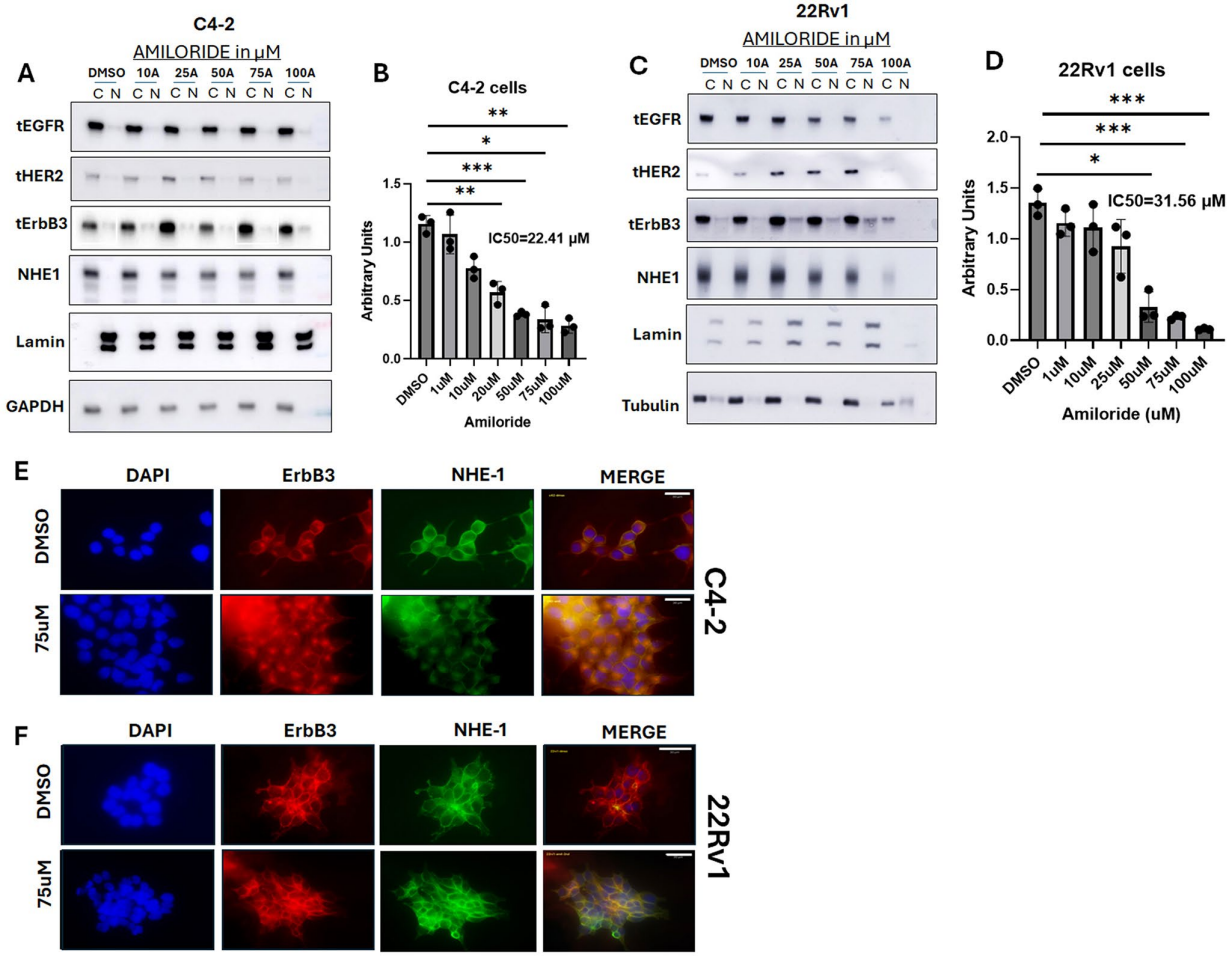
Amiloride does not promote ErbB3 translocation from the nucleus to the cytoplasm and the plasma membrane in CRPC cells (**A**) Hormone-insensitive C4-2 or (**C**) the unrelated cell line 22Rv1 cells were also treated with varying concentrations of amiloride for 72 h before being subjected to viability assays or lysed and fractionated as previously described. For all viability assays, results were obtained from triplicate experiments. Error bars represent standard deviation. Tables show p-values with respect to DMSO for each tested cell line. All densitometry was performed using ImageJ. C = cytoplasmic and N = nuclear. (**B**, **D**) Cells were subjected to viability assays using the stated concentrations of amiloride. p-values are calculated with respect to DMSO. (**E**, **F**) C4-2 and 22Rv1 cells were treated with IF-specific antibodies to NHE-1 (prototypical target of amiloride) and imaged as described in previous figure legends

In contrast, in C4-2 cells, all ErbB receptor tyrosine kinases, including ErbB3, were overwhelmingly cytoplasmic, even at baseline (Fig. 2A). Cell viability still showed a dose-dependent decrease with increasing amiloride concentration causing half-maximal inhibition at 22.41 µM, indicating a different mode of action of amiloride in these cells (Fig. 2B). When the CRPC cell line 22Rv1 was similarly examined, there was a dose-dependent decrease in EGFR, but a dose-dependent increase in HER2 with increasing amiloride, while ErbB3 levels were not significantly affected (Fig. 2C). As a result, 22Rv1 cells were significantly less susceptible to amiloride (Fig. 2D), and indicate correlation between sensitivity to amiloride and ErbB3 localization. However, in both C4-2 cells (Fig. 2E), as well as in 22Rv1 (Fig. 2F), amiloride treatment increased ErbB3 localization to the plasma membrane– as indicated by co-localization with NHE1. Taken together, these results indicate that the cytotoxic effects of amiloride correlates with its translocation from the nucleus to the cytoplasm in LNCaP and from the cytoplasm to the plasma membrane in the CRPC lines.

### The tyrosine kinase HER2, that regulates the activation of downstream targets, is itself activated by the ErbB3 ligand HRG1

**Fig. 3:**
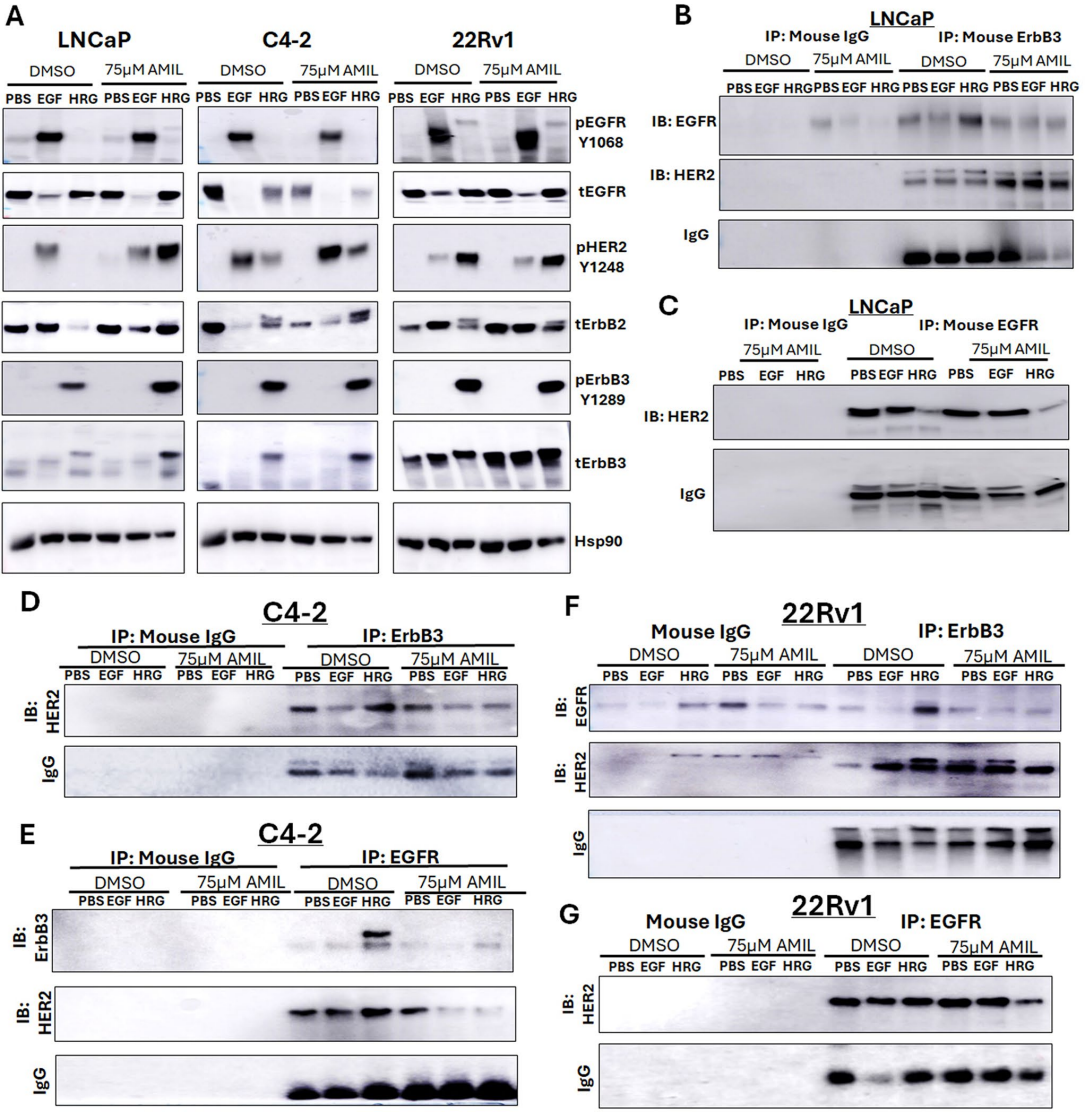
Differential activation and dimerization of ErbB family members and their downstream targets in HSPC and CRPC cells with high concentrations of amiloride (**A**) HSPC (LNCaP) and CRPC (C4-2, 22Rv1) cells were treated for 72 h with 75µM amiloride dissolved in 100% sterile DMSO and stimulated with PBS, EGF or HRG for 15 min prior to collection to observe activation of ErbB family members and their downstream targets. Cells were lysed in denaturing lysis buffer before being analysed by immunoblotting. 25 µg of protein were loaded per lane. Hsp90 was used as a loading control. (**B**,**C**) LNCaP cells (HSPC) or (**D**,**E**) C4-2 cells (CRPC) or (**F**, **G**) 22Rv1 cells (unrelated CRPC cells) were treated with 75µM amiloride or 100% DMSO (0.1% v/v) for 72 h and stimulated with PBS, EGF or HRG for 15 min to activate ErbB family dimers just prior to collection. 400ug of whole cell lysate were used in each pulldown lane. Mouse IgG antibody was used as an isotype control. Amiloride increases ErbB3-HER2 dimers and stabilizes ErbB3-EGFR dimers

We next investigated the effects of amiloride on the phosphorylation status of the EGFR family and their prominent downstream targets, considered a measure of activation of these proteins. Transcript levels of any of the ErbB family members were largely unchanged across cell lines, except for EGFR mRNA in 22Rv1 cells which increased with 75µM amiloride (Supp. Figure 1A). We previously showed that the ErbB3 ligand HRG1, but not the EGFR ligand EGF, stimulated ErbB3 nuclear translocation [24]. Hence, we probed the effect of both EGF and HRG1 on ErbB receptor phosphorylation that corresponded to their activation in the presence or absence of 75µM amiloride. EGFR activation was determined by its phosphorylation at Y1068, a Grb2 binding site. EGF, but not HRG1, induced EGFR phosphorylation, and this effect was not altered by amiloride in any of the cell lines investigated (Fig. 3A). In LNCaP cells, HER2 phosphorylation at Y1248 was, however, stimulated by EGF under control conditions, whereas HRG1 activated it only upon amiloride treatment. In the CRPC lines, however, it was stimulated by both EGF as well as HRG1, but amiloride did not affect this distribution (Fig. 3A). On the other hand, in all three cell lines, ErbB3 phosphorylation at Y1289 was stimulated by HRG1 but not EGF.

Next, we investigated the downstream targets of the ErbB receptors. Both ERK and Akt are activated by all four receptors, but ErbB3 is more likely to activate Akt, given that it has six PI3K^p85^ binding sites (PI3K being upstream of Akt), compared to EGFR and ErbB2 [12, 13, 15]. Similar to ErbB3 activation, Akt phosphorylation was induced by HRG1 mostly in the presence of amiloride in LNCaP cells, whereas in C4-2 and 22Rv1 cells, it was activated both in the presence and absence of amiloride (Supp. Figure 1B). In contrast, ERK was phosphorylated mostly in the same pattern as HER2 phosphorylation (Supp. Figure 1B). Taken together, these results suggest that EGF activates EGFR, HRG1 activates ErbB3, but both EGF and HRG activate HER2 and downstream targets. Since HER2 does not have a known ligand, its activation indicates heterodimerization with EGFR or ErbB3.

### Amiloride selectively promotes HER2/ErbB3 heterodimerization and inhibits HRG1-induced EGFR/ErbB3 heterodimers

We next investigated whether amiloride-mediated activation of downstream EGFR family targets could be explained by receptor dimerization patterns. In LNCaP cells, HRG1, but not EGF, stimulated EGFR/ErbB3 dimerization, that was suppressed by amiloride (Fig. 3B) whereas amiloride significantly increased HER2/ErbB3 dimerization independent of ligand binding (Fig. 3B). In contrast, EGFR/HER2 dimers were diminished upon HRG1 stimulation independent of the presence of amiloride (Fig. 3C), suggesting that under control conditions, HRG1 enabled realignment of EGFR from HER2 binding to ErbB3 binding, whereas upon amiloride treatment, the EGFR/ErbB3 dimers were diminished, while HER2/ErbB3 dimers were stabilized (Fig. 3C). This is likely due to the decrease in EGFR protein levels with 75 µM treatment observed in Fig. 1A in these cells. Loss of EGFR/ErbB3 levels upon high dose amiloride treatment likely explains why at even higher doses (100 µM), nuclear ErbB3 is restored despite increased EGFR levels at that dose.

Similar to LNCaP, C4-2 cells also saw increased HRG-activated EGFR-ErbB3 dimers that were suppressed by amiloride treatment (Fig. 3D) but in these cells, there was a decrease in HER2/EGFR dimers with amiloride treatment both in the presence of EGF and HRG1 (Fig. 3D). In these cells, HER2/ErbB3 was reduced by EGF treatment, but not by HRG1 (Fig. 3E) suggesting realignment of HER2 from ErbB3 to EGFR upon EGF stimulation, that was reversed by amiloride. Amiloride promoted co-localization of HER2 with ErbB3 in these cells is better illustrated by immunofluorescent imaging (Supp. Figure 2).

In 22Rv1, again, HRG1 induced EGFR/ErbB3 dimerization, that was suppressed by amiloride (Fig. 3F), which also stabilized HER2/ErbB3 dimerization (Fig. 3F), but showed no significant change in EGFR/HER2 dimers (Fig. 3G), perhaps due to the very high levels of EGFR expression in these cells (Supp. Figure 1A), in contrast to LNCaP, where amiloride suppresses EGFR protein levels, perhaps due to a post-translational modification step. Thus, a comparison of HSPC LNCaP cells with CRPC C4-2 and 22Rv1 cells shows that in both HSPC and CRPC lines, HRG1 realigned EGFR from HER2 to ErbB3, whereas in CRPC lines, EGF realigned HER2 from ErbB3 to EGFR. In all three lines, HRG1 stimulated EGFR/ErbB3 dimerization, whereas amiloride prevented this effect, while promoting HER2/ErbB3 dimers.

### Effect of HER2 receptor tyrosine kinase on ameliorating C4-2 cell viability is enhanced by amiloride treatment

**Fig. 4:**
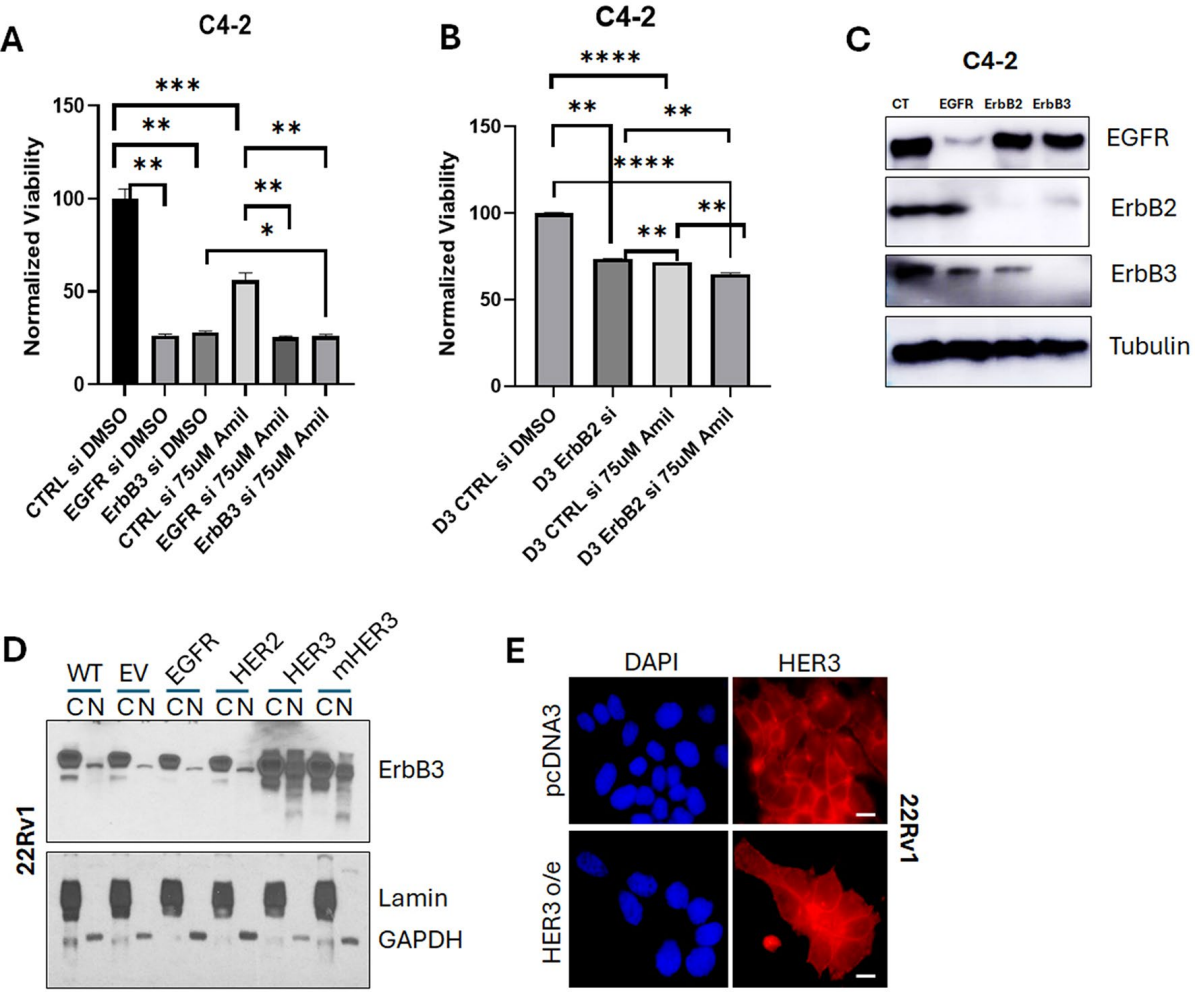
Amiloride efficacy is enhanced by EGFR knockdown in HSPC cells and by HER2 knockdown in CRPC cells. (**A**) CRPC C4-2 cells were transfected with control (CT) or EGFR or ErbB3 siRNA or (**B**) HER2 siRNA and treated with or without 75µM amiloride before being analysed for changes in viability with the MTT assay. Error bars represent standard deviation. Experiments were performed in triplicate. (**C**) Whole cell immunoblot for siRNA efficacy. 20 µg of protein were loaded per lane. Tubulin was used as a loading control. (**D**) 22Rv1 cells (CRPC) were transiently transfected with empty vector (EV), EGFR (B1), ErbB2 (B2), ErbB3 (B3) or mutant ErbB3 (mB3) as previously described by us in detail [23, 45]. Cells were collected, fractionated and analysed by immunoblot as described in previous figure legends. (**E**) ErbB3 overexpression was visualized microscopically in 22Rv1 cells using the reagents and procedures as described in previous figure legends

We next investigated whether silencing the receptors would decrease cell viability. We used EGFR-, HER2- or ErbB3-specific silencing RNA (siRNA) sequences at a concentration of 10 pM per treatment condition to determine whether EGFR family receptors were involved in decreasing cell viability in response to amiloride treatment. The efficacy and specificity of EGFR family silencing has been previously assessed by us [23]. We used CRPC C4-2 cells to test the effect of EGFR family on mediation of the effects of this drug. C4-2 cells showed significant decreases in viability with EGFR and ErbB3 siRNAs individually (approximately 75% decrease in viability using each siRNA) (*p* = 0.0015) (Fig. 4A). As before, amiloride significantly inhibited C4-2 cell growth, but knockdown of EGFR in the amiloride treated cells had no further effect, although ErbB3 knockdown slightly decreased viability (*p* = 0.0321) (Fig. 4A). However, knockdown of HER2 in C4-2 cells reduced viability by about 30% (*p* < 0.0001), whereas in amiloride treated cells, the same knockdown reduced viability by an additional 10% (*p* = 0.0041) (Fig. 4B). The efficacy of the siRNAs to EGFR, HER2, ErbB3 in C4-2 cells is shown in Fig. 4C. Thus, treatment with amiloride increased sensitivity to HER2 knockdown, and explains the important role of this receptor in CRPC cell viability.

### Overexpression of ErbB3 promotes its nuclear localization

Since HRG1 appears to promote dimerization of HER2 with ErbB3, and the impact of nuclear ErbB3 on this dimerization, we next investigated whether overexpression of ErbB3 would affect its nuclear localization. EGFR, HER2 and HER3 was overexpressed in 22Rv1 cells as indicated (Fig. 4D). We used 22Rv1 cells as these cells– unlike LNCaP which showed strong nuclear staining or C4-2 cells, which showed almost no nuclear staining, demonstrated small amounts of nuclear ErbB3. Overexpression of HER3/ErbB3 caused significant increase in nuclear ErbB3 levels (Fig. 4D), indicating that *de novo* ErbB3 expression results in nuclear localization, whereas it then has to be transported to the cytoplasmic compartment. These observations are supported by immunofluorescent pictures showing that overexpression of ErbB3 in 22Rv1 cells resulted in nuclear expression (Fig. 4E). To determine whether phosphorylation of ErbB3 at major tyrosine kinase sites (Y1222, Y1289, and Y1328) contributed to its nuclear localization, we mutated these sites to alanine and overexpressed the mutant plasmid (mHER3). There was no change in nuclear ErbB3 expression indicating that phosphorylation of ErbB3 did not contribute to its nuclear localization. Taken together, the results shown so far indicate that (i) EGFR and HER2 were mainly plasma membrane located in PCa cells while ErbB3 may localize on the membrane, the cytoplasm or the nucleus; (ii) amiloride promoted ErbB3 translocation from the nucleus to the cytoplasm and the cytoplasm to the plasma membrane; (iii) as a result, amiloride enhanced HER2/ErbB3 dimerization, (iv) HER2 was activated by dimerization with ErbB3 and (v) activated HER2 enhanced downstream signaling and cell proliferation.

### Amiloride enhances the sensitivity of HSPC cells to lapatinib

We have previously shown that physiological concentrations of the FDA-approved reversible HER2 kinase inhibitor lapatinib were ineffective in inhibiting the growth of PCa cells [23]. Based on the data above, we hypothesized that the addition of amiloride to lapatinib would enhance cell killing efficacy, at physiological doses of both drugs. We therefore investigated the effects of 10 µM amiloride, the least dose usually used in cell lines [47] on sensitizing PCa cells to lapatinib.

**Fig. 5:**
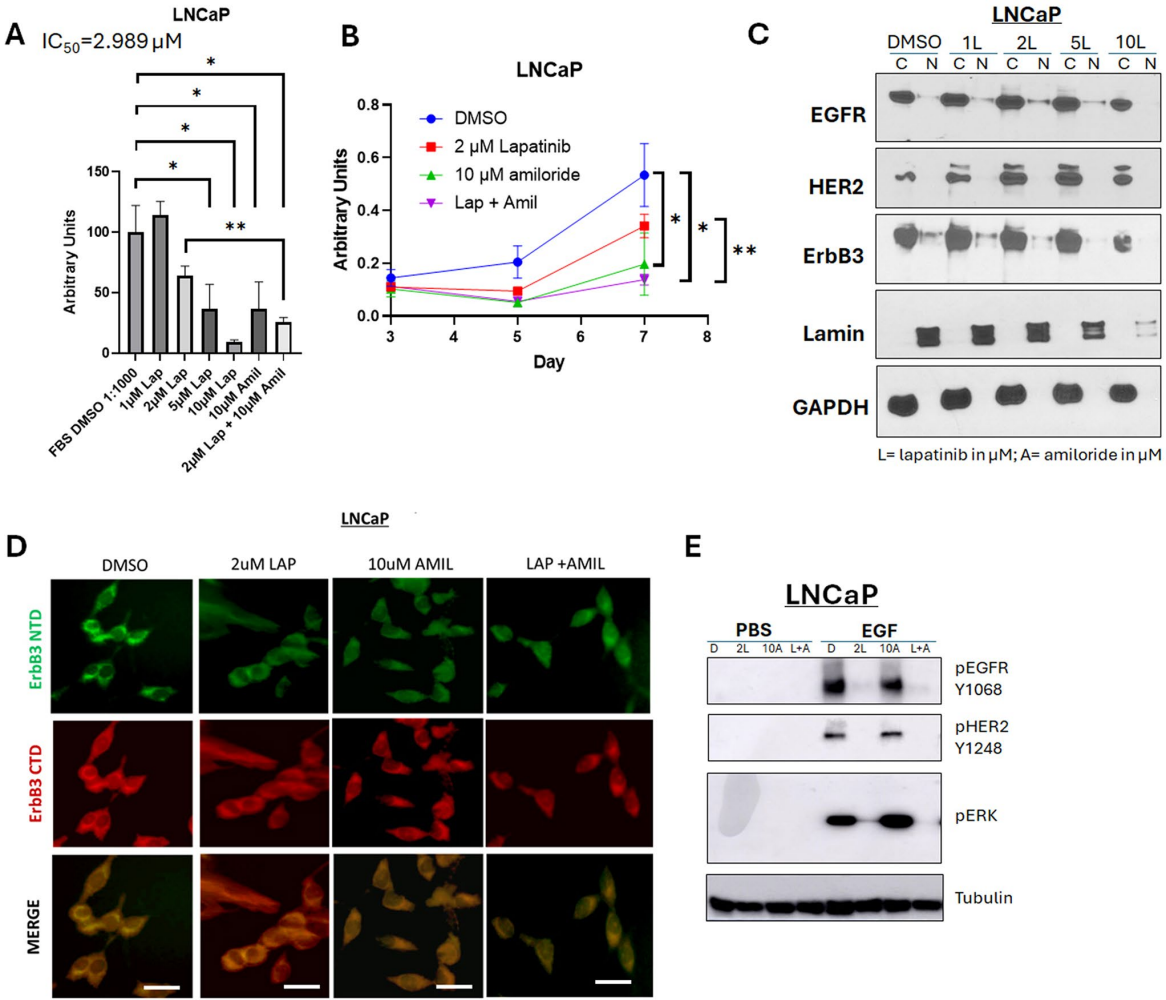
Amiloride enhances the sensitivity of HSPC cells to low concentrations of lapatinib (**A**, **B**) LNCaP cells were treated with 1–10µM lapatinib and assayed for viability. Lapatinib and amiloride were both dissolved in 100% DMSO. In a head-to-head comparison of lapatinib and amiloride, the combination was additive when cell viability was assayed with the MTT reagent. Experiments were done in triplicate. Error bars represent standard deviation. (**C**) Cells were treated with varying concentrations of lapatinib for 72 h before being collected and lysed into cytoplasmic and nuclear fractions as described in earlier figure legends. (**D**) Cells were treated with 2µM lapatinib, 10µM amiloride or a combination of the two for 72 h before being collected, fixed and processed for indirect immunofluorescent microscopy using immunofluorescent-specific antibodies to the C- and N- termini of ErbB3 (‘CTD’ and ‘NTD’ respectively) as previously described. Scale bars = 7.5 μm. Co-administration of lapatinib and amiloride increases the accumulation of ErbB3 compared to 2µM lapatinib alone. (**E**) LNCaP cells were treated for 72 h with lapatinib, amiloride or the combination or 100% sterile DMSO and stimulated with PBS, EGF or HRG for 15 min prior to collection to observe activation of ErbB family members and their downstream targets. Cells were lysed in denaturing lysis buffer before being analysed by immunoblotting. 25 µg of protein were loaded per lane. Tubulin was used as a loading control

We previously demonstrated that 2 µM lapatinib was the least dose that was effective in PCa cells [23]. We investigated the viability of LNCaP cells treated with increasing concentrations of lapatinib and established an IC_50_ = 3 µM (Fig. 5A); hence we used 2 µM as a suboptimal dose, low dose amiloride (10µM) or a combination of 2µM lapatinib and 10µM amiloride (‘2µM Lap + 10µM Amil’) to determine whether amiloride enhanced the efficacy of lapatinib in these cells. The viability of LNCaP cells decreased only 36% with 2µM lapatinib (*p* = 0.0925) compared to 90.5% at the highest concentration of lapatinib tested (10µM, *p* = 0.0192). 10µM amiloride individually produced a reduction of 63.2% (*p* = 0.0252). When 2 µM lapatinib was combined with 10 µM amiloride, the resulting decrease in viability was 74.2% (*p* = 0.0259) which was comparable to 10 µM lapatinib alone (*p* = 0.0091) (Fig. 5B).

When the expression and localization of total protein levels of EGFR family members were analyzed, little change was seen in EGFR or HER2 with lapatinib (Fig. 5C). In contrast, ErbB3 nuclear localization decreased in a dose-dependent manner from 0–10µM lapatinib (Fig. 5C). At 2 µM lapatinib, though, sufficient levels of ErbB3 remained in the nucleus. To overcome this, immunofluorescent analysis of ErbB3 localization using an anti-C-terminal (CTD) and an anti-N-terminal (NTD) ErbB3 antibody depicted nuclear localization of ErbB3 under control conditions (Fig. 5D). 2 µM lapatinib or 10µM amiloride did not disturb this pattern (Fig. 5D). However, immunofluorescent imaging showed that with 2µM lapatinib treatment in the presence of amiloride, ErbB3 localization was cleared from the nucleoplasm. An immunoblot analysis of the signaling cascades under all the treatment conditions revealed that EGFR underwent phosphorylation (or activation) with EGF treatment, ErbB3 was phosphorylated with HRG1 and HER2 with both (Fig. 5E). With 10 µM amiloride, unlike 75 µM, there was no change in EGF-induced EGFR and HER2 phosphorylation or in HRG1-induced ErbB3 phosphorylation, but HRG1-induced HER2 phosphorylation was severely affected (Fig. 5E). Significantly, lapatinib treatment, with or without amiloride, abrogated EGFR and HER2 phosphorylation in LNCaP cells but ERK phosphorylation was eliminated only with the combination (Fig. 5E).

**Fig. 6:**
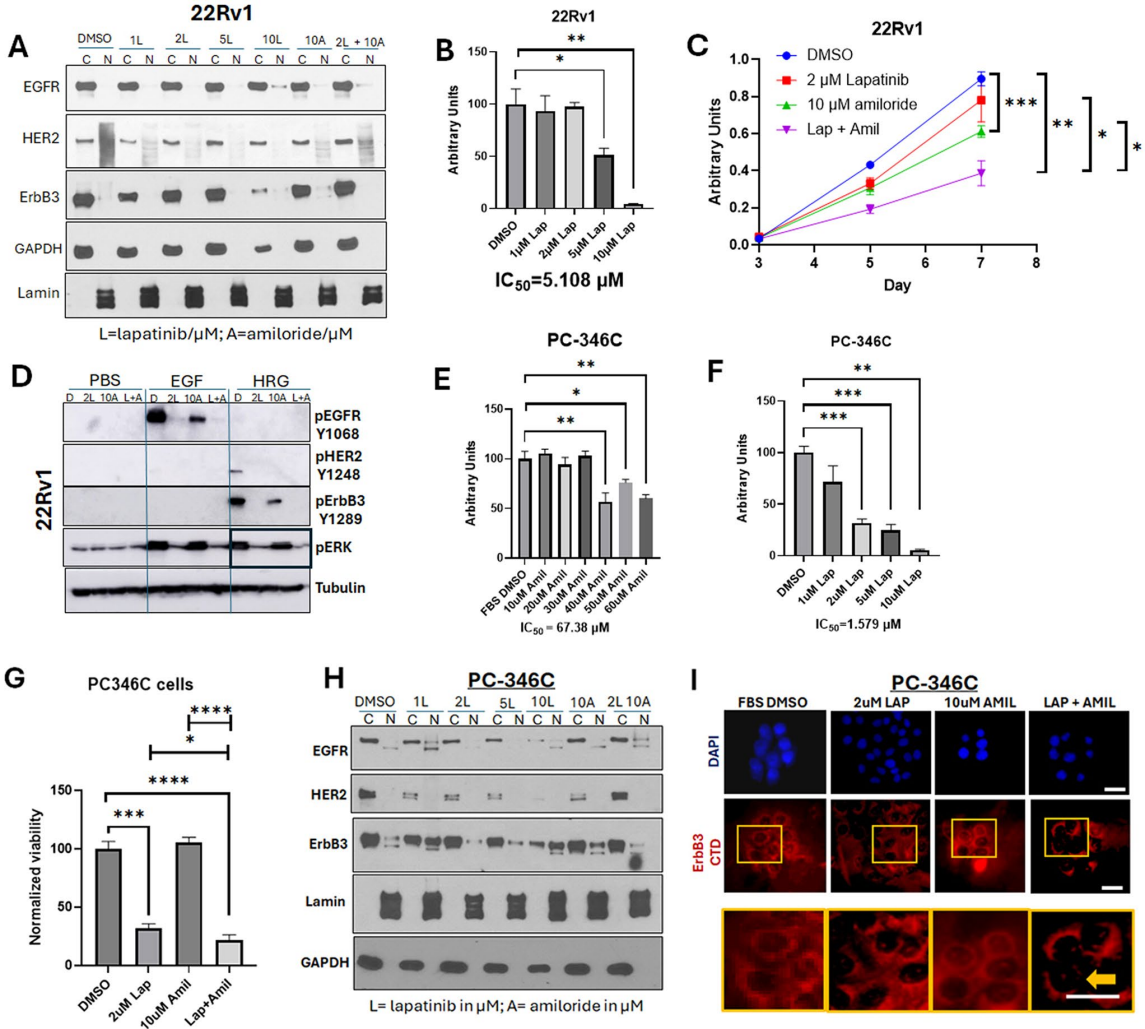
Amiloride enhances the sensitivity of HSPC and CRPC cell lines to low concentrations of lapatinib. (**A**) 22Rv1 cells were treated with varying concentrations of lapatinib, 10µM amiloride or a combination of the two for 72 h before being collected and lysed into cytoplasmic and nuclear fractions as described in earlier figure legends. Co-administration of lapatinib and amiloride does not increase the accumulation of ErbB3 in the cytoplasmic fraction. (**B**) 22Rv1 cells were treated with 1–10µM lapatinib and assayed for viability. Lapatinib was dissolved in 100% DMSO. Experiments were done in triplicate. Error bars represent standard deviation. (**C**) In a head-to-head comparison of lapatinib and amiloride, the combination was additive when cell viability was assayed with the MTT reagent. Experiments were done in triplicate. Error bars represent standard deviation. Coloured dotted line estimates 50% viability. Table shows p-values with respect to FBS DMSO. (**D**) 22Rv1 (CRPC) cells were treated for 72 h with lapatinib, amiloride or the combination or 100% sterile DMSO and stimulated with PBS, EGF or HRG for 15 min prior to collection to observe activation of ErbB family members and their downstream targets. Cells were lysed in denaturing lysis buffer before being analysed by immunoblotting. 25 µg of protein were loaded per lane. Tubulin was used as a loading control. Tables show p-values with respect to PBS DMSO in each cell line tested. (**E**) PC-346 C cells were treated with 10–60 µM of amiloride and assayed for viability. Amiloride was dissolved in 100% DMSO. Experiments were done in triplicate. Error bars represent standard deviation. (**F**) PC-346 C cells were treated with 1–10µM lapatinib and assayed for viability. Lapatinib was dissolved in 100% DMSO. Experiments were done in triplicate. Error bars represent standard deviation. (**G**) PC-346 C cells were treated with lapatinib, amiloride or the combination, which is shown to be additive when cell viability was assayed with the MTT reagent. Experiments were done in triplicate. Error bars represent standard deviation. (**H**) PC-346 C cells were treated with varying concentrations of lapatinib, 10µM amiloride or a combination of the two for 72 h before being collected and lysed into cytoplasmic and nuclear fractions as described in earlier figure legends. (**I**) Cells were treated with 2µM lapatinib, 10µM amiloride or a combination of the two for 72 h before being collected, fixed and processed for indirect immunofluorescent microscopy using immunofluorescent-specific antibodies to the C-terminal domain of ErbB3 as previously described. Yellow boxes (inset) and bold yellow arrow depict area of negligible ErbB3 nuclear staining. Scale bars = 7.5 μm

In contrast to LNCaP cells, in 22Rv1 cells, which had very low baseline levels of nuclear ErbB3, this RTK remained cytoplasmic with lapatinib treatment, as well as with amiloride combinations, similar to EGFR and HER2 (Fig. 6A). These cells did however show a dose-dependent decrease in viability from 0–10µM lapatinib, with a 49% decrease with 5 µM lapatinib (*p* = 0.0157) and a 96% decrease at 10 µM lapatinib (*p* = 0.0075) with a resultant IC_50_ of 5.108 µM (Fig. 6B**).** However, the 10 µM intratumoral dose will not be physiologically relevant since achievement of that dose will put patients in conditions that will subject them to various adverse events prior to achieving that dose. To determine whether the more physiological dose of 2 µM can be enhanced by the addition of amiloride, we tested the combination of the two drugs in 22Rv1 cells (Fig. 6C). As before, 10 µM amiloride (that will not cause hyperkalemia) had by itself a small effect on the viability of 22Rv1 cells (*p* = 0.0007); however– the combination of 2 µM lapatinib and 10 µM amiloride reduced 22Rv1 viability by 49.9% (*p* = 0.0012), and the combinatorial effect was more significant in this cell line (than in LNCaP cells) in comparison to either lapatinib alone (*p* = 0.0131) or amiloride (*p* = 0.0164). Although there was no significant effect on AR transcriptional activity at these drug concentrations (Supplementary Fig. 3A), lapatinib significantly suppressed the activation of the RTKs and their downstream targets, with or without the presence of amiloride (Fig. 6D). Significantly, as in LNCaP cells, ERK phosphorylation was significantly decreased in HRG1-stimulated 22Rv1 cells upon combinatorial treatment, compared to lapatinib alone (Fig. 6D). while amiloride but not lapatinib eliminated any nuclear ErbB3 that still may remain in these cells (Supplementary Fig. 3B). Lapatinib (2 µM) and amiloride (10 µM) together showed a combinatorial effect when plotted against increasing doses of each drug individually (Supplementary Fig. 3C).

Thus far, we have used three cell lines– that are sensitive to amiloride (IC_50_ in the range 20–40 µM), however, we then investigated the effect of the combination on PC-346 C cells, previously reported by us [12] that is more resistant to amiloride [IC_50_ = 67.38 µM (55.26 µM -108.3 µM)] (Fig. 6E). These cells express wild type AR at very low levels and are considered to be hormone sensitive since they are inhibited by flutamide [48]. We therefore tested the effect of the amiloride-lapatinib combination on PC-346 C cells, which are, however, very sensitive to lapatinib [IC_50_ = 1.579 µM (1.203 µM -2.001 µM)] (Fig. 6F). While 10 µM amiloride had no effect on PC-346 C cells, 2 µM lapatinib caused a 68% decrease in viability (*p* = 0.0003) (Fig. 6F). The combination of lapatinib and amiloride caused an additional 32.5% decrease in viability (*p* = 0.0440 compared to lapatinib alone) (Fig. 6G), indicating a combinatorial effect even in these amiloride-resistant cells.

To determine whether the mechanism by which the combination works in a second hormone-sensitive PCa cell line PC-346 C [49] is similar to that in LNCaP cells, we tested the effects of these treatments on EGFR, HER2 and ErbB3. Like the other lines, EGFR and HER2 was mostly cytoplasmic, and remained so, irrespective of the treatment. ErbB3 was partly nuclear, and the nuclear expression was enhanced by amiloride treatment, which explains its resistance to this drug (Fig. 6G). In contrast, lapatinib alone did not affect ErbB3 nuclear levels, but in the presence of amiloride, significantly reduced ErbB3 nuclear localization further, explaining the additive effect on cell viability (Fig. 6H). This is reinforced by immunofluorescent imaging showing that the combination of lapatinib and amiloride removes the levels of nuclear ErbB3 (Fig. 6I). Taken together, in hormone sensitive cells, the presence of nuclear ErbB3 induces resistance to cell death, whereas treatment with lapatinib and amiloride reduces ErbB3 nuclear localization and reduces viability.

### Low dose amiloride and lapatinib combine to induce apoptosis in HSPC cells

**Fig. 7:**
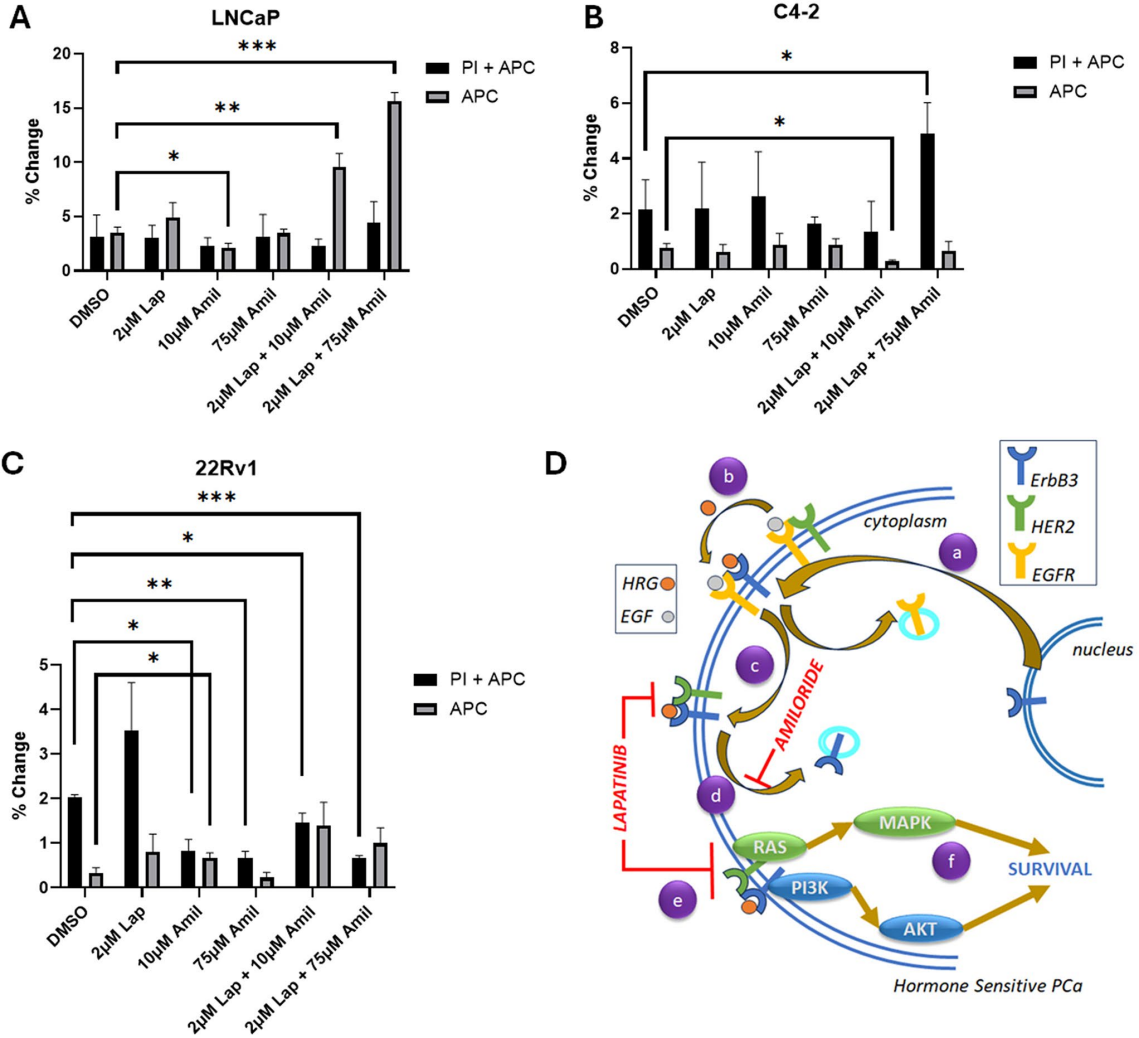
Amiloride and lapatinib synergize to increase apoptosis in HSPC and CRPC cell lines. (**A**-**C**) HSPC and CRPC cell lines were treated with 100% DMSO, lapatinib, amiloride or the combination (in µM) for 72 h before being processed for cell death analysis using annexin V and propidium iodide staining. The percentage of cells undergoing early or late apoptosis with DMSO treatment was set to 100% and values for the various treatment conditions calculated accordingly. Experiments were performed in triplicate. Error bars represent standard deviation. (**D**) Schematic with proposed molecular mechanism of lapatinib-amiloride efficacy. (**a**) EGFR, HER2 and ErbB3 exist at the cell membrane and signal via pathways such as ERK and AKT. (**b**,**c**) ErbB3 monomers cycle between the nucleus and cell membrane. (EGFR and HER2 behave similarly but have been omitted for clarity). (**d**) Lapatinib is a dual-kinase TKI (tyrosine kinase inhibitor) of HER2 and EGFR dimers but will also inhibit HER2 in HER2-ErbB3 dimers. Lapatinib is unlikely able to inhibit ErbB3 if it is in the nucleus and not at the cell surface. (**e**) Amiloride is a macropinocytosis inhibitor that prevents internalization of ErbB3 and retains it at the cell surface. As a result, nuclear ErbB3 decreases and cytoplasmic surface ErbB3 increases. (**f**) Amiloride-induced ErbB3 retention enables its dimerization with HER2, enabling the formation of ErbB3-HER2 dimers which are now inhibited by the addition of low concentrations of lapatinib

The goal of cancer treatment is to ensure that all malignant cells are dead, not dormant. However, the changes in cell viability that we have conducted thus far could be due to an increase in apoptosis, or the onset of various mechanisms that may have led to cellular quiescence. Hence, we conducted cell death analyses to ascertain the mechanism causing the consistent decreases in viability seen in all 3 cell lines with the combination of low dose lapatinib and amiloride. Flow cytometry was employed using DNA-bound propidium iodide (PI) as a necrosis marker and cell surface expression of Annexin V using the Annexin V-Allophycocyanin (APC) conjugate as a marker of apoptosis. LNCaP cells showed no significant change in early apoptosis (‘APC’) or late apoptosis (apoptosis with necrosis, ‘PI + APC’) with 2µM lapatinib and a slight decrease in early apoptosis (-39.6%) with 10µM amiloride (*p* = 0.023) (Fig. 7A). The combination of 2µM Lap + 10µM amiloride produced a sharp increase in the percentage of cells undergoing early apoptosis (2.7-fold, *p* = 0.0061) and this was increased further when amiloride was used at 75µM (4.42-fold, *p* < 0.0001), although no change in the fraction of cells in late apoptosis was noted (Fig. 7A). In contrast, C4-2 cells exhibited an increase in necrotic cells only when 2 µM lapatinib + 75µM amiloride were used (2.26-fold, *p* = 0.0371) which may indicate toxicity, rather than programmed cell death, while a combination of 2 µM lapatinib + 10µM amiloride actually resulted in a 65% decrease in apoptosis (*p* = 0.0193) (Fig. 7B). Similar to C4-2 cells, 22Rv1 cells displayed no change in apoptosis when exposed to 2µM lapatinib either alone or in combination with 10 µM or 75 µM amiloride, and significantly reduced cell death with 2µM lapatinib in combination with both 10µM and 75µM amiloride (Fig. 7C). Representative raw readings for these data are provided in supplementary information (Supp. Figs.  4, 5 and 6). Thus, in LNCaP cells, the decrease in cell viability with the combination of 2 µM lapatinib + 75µM amiloride observed is likely due to an increase in apoptosis while that in C4-2 cells, any change in viability is likely caused by increase in toxicity, and no substantial effects of the combination was observed in 22RV1 cells. Taken together, this indicates that the combination of 2 µM lapatinib + 10µM amiloride was effective in inducing programmed cell death in HSPC LNCaP cells (higher doses may cause toxicity), but not in CRPC lines at any dose.

## Discussion

In this paper we present a novel treatment strategy for inhibiting HSPC using amiloride to enhance the cytotoxicity of the FDA-approved reversible dual TKI lapatinib. Lapatinib possesses several advantages as a therapeutic strategy in PCa– it is well-established, relatively well tolerated (with mild diarrhea and rash being the most common toxicities) and is administered orally [22]. Major pathways involved in its efficacy and resistance have been identified and investigated [50]. Lapatinib inhibits the kinase domains of HER2 and EGFR following their dimerization [51], preventing receptor auto- and transphosphorylation and subsequent activation of downstream pathways, for example ERK/MAPK [52]. Lapatinib was shown to bind to lipid membranes and insert into the lipid-water interface of the lipid bilayer [53]. It has been well-documented that lapatinib is most effective on HER2/ErbB3 dimers. Lapatinib induces HER2/ErbB3 dimers [54]; while increased HRG1 and activated ErbB3 strongly correlated with lapatinib sensitivity [55]. Therefore, it is necessary for HER2 to dimerize with ErbB3 in the plasma membrane for the heterodimer to be accessible to lapatinib for efficacy.

Here, we demonstrate that while ErbB3 can translocate between the plasma membrane, the cytosol and the nucleus, HER2 was primarily located in the plasma membrane, in support of our previous observations [24]. However, for the HER2/ErbB3 dimer to form, ErbB3 would need to be located on the plasma membrane. We demonstrate that amiloride causes a dose dependent translocation of ErbB3 from a nuclear to a cytoplasmic/membranous location in HSPC LNCaP cells. Investigation of the phosphorylation status of these RTKs demonstrated that EGF activates EGFR, HRG1 activates ErbB3, but both EGF and HRG activate HER2 and downstream targets, while HRG1-induced HER2 phosphorylation is enhanced by amiloride mainly in HSPC cells. Co-immunoprecipitation and immunofluorescence studies suggested that HRG1 realigned EGFR from HER2 to ErbB3, while EGF realigned HER2 from ErbB3 to EGFR\. Additionally, amiloride realigned HER2 from EGFR to ErbB3 containing dimers in HSPC cells; thus, stabilizing the HER2/ErbB3 heterodimers in the cytoplasmic/membranous fraction.

Knockdown of the RTK genes demonstrated that amiloride enhanced the cell killing properties associated with HER2 knockdown. Hence, we used the HER2 inhibitor lapatinib to enhance the loss of viability induced by amiloride. The combination enabled the use of the drugs at much lower doses to prevent adverse cellular toxicity. We demonstrated that in LNCaP cells, but not in CRPC cells, this loss of viability was caused by an increase in apoptosis. Taken together, these results indicate that amiloride induces apoptosis in HSPC cells by enabling HER2/ErbB3 accumulation in the cytoplasmic/membranous domain, where it can be inhibited by lapatinib (Fig. 7D).

An interesting phenomenon noted is that in all three cell lines HRG1 stimulation, but not EGF stimulation, induces EGFR/ErbB3 heterodimerization, which is inhibited by high dose amiloride. In LNCaP cells, this increase in EGFR/ErbB3 comes at the expense of EGFR/HER2 dimers, but this stark contrast is not observed in the other cell lines. The cause for this observation Is perhaps explained by the expression levels of the ErbB receptors in the three cell lines shown in Supplementary Fig. 1A. LNCaP cells express limited levels of the three receptors and presumably all receptors are dimerized; hence, when HRG1 stimulates ErbB3, the activated receptor destabilizes EGFR/HER2 dimers to promote EGFR/HER3. In CRPC cells, especially in 22Rv1, on the other hand, EGFR levels were so high that both EGFR/HER2 and EGFR/HER3 heterodimers can be accommodated. At the same time, amiloride stabilizes HER2/ErbB3 heterodimers irrespective of stimulation, especially in LNCaP. Thus, in these cells, amiloride realigned ErbB3 from EGFR-containing dimers to HER2 containing dimers, thereby stabilizing HER2/ErbB3 heterodimerization. HER2/ErbB3 is a preferred heterodimer and constitutes a high affinity co-receptor for HRG1 [56]. This dimer potently activates both the phosphatidylinositol 3-kinase (PI3K)/Akt and Ras/mitogen activated protein kinase (MAPK) pathways. More importantly, this dimer is a target of lapatinib and is acted upon by the latter mainly in the plasma membrane.

Lapatinib has previously been used at 10 µM by other labs [57] while we have used it at 2 µM [23]. Although other labs have reported an IC_50_ of 0.48 µM and 0.36 µM for lapatinib in LNCaP and PC-3 cells, respectively after 24 h of treatment [58], in our hands, the IC_50_ levels obtained after 7 days of treatment were much higher; however, we have maintained analysis of viability for up to 7 days and analysis of protein expression at 72 h to enable determination of long-term effects of the drug under investigation. Amiloride has been used at doses up to 1 mM in cell lines [30, 46]. The recommended dose of amiloride is 10 mg twice daily for adults with hypertension or congestive heart failure. If no response is seen, the recommendation is to increase the dose every 4 days in increments of 10 mg twice daily to a maximum dosage of 30 mg twice daily. However, amiloride has very low toxicity; at 20–25 times the recommended dose, it causes hypokalemia; however, this risk is reduced 10-fold by concomitant intake of a kaliuretic thiazide diuretic. High amiloride doses are required in cancer management due to poor absorption; to counter this, multiple amiloride variants have been developed that can be used at much lower doses [59]. We will use similar compounds for preclinical development, but the current manuscript is solely focused on the mechanism of its effects on ErbB3 localization.

Here, we have used amiloride at two doses– low (10 µM) and high (75 µM), which are well below the doses used earlier in cell lines and underscore the efficacy of the drug in PCa cells. High concentrations of amiloride dose-dependently decreased nuclear ErbB3 and increased cytoplasmic ErbB3 in HSPC cells. The CRPC lines C4-2 and 22Rv1 did not display this effect as basal ErbB3 was predominantly cytoplasmic in these cells. Indeed, in 22Rv1 cells we observed a dose-dependent reduction in cytoplasmic EGFR protein but a compensatory increase in transcript levels, likely suggesting greater post-translational degradation [60].

The combination of Lap + Amil decreased cell viability by significantly increasing early apoptosis. We noted with interest that in LNCaP cells, apoptosis was observed when ErbB3 was entirely cytoplasmic, and its nuclear expression was minimal. From this we reason that lapatinib increases cytoplasmic/membranous HER2 while amiloride causes ErbB3 cytoplasmic/ membranous accumulation as well. ErbB3 thus confined to the plasma membrane dimerizes with HER2, enabling the formation of active ErbB3/HER2 dimers, whose kinase domains are now targeted by lapatinib, whose primary mechanism of action is inhibition of cytoplasmic/membranous HER2/ErbB3 dimers. Thus, we conclude that amiloride followed by lapatinib would likely reduce tumor volume selectively in HSPC tumors, and therefore may be studied as neoadjuvant therapy to improve outcomes from RP.

Amiloride induced ErbB3 localization to the plasma membrane is likely unrelated to its documented effect on members of the NHE family or on ENaC. Koumakpayi et al. [30] have proposed a role for amiloride in ErbB3 endocytosis; likely related to its NHE activity [33] where they hypothesize that endocytosis leads to nuclear localization of ErbB3 and that amiloride, as a macropinocytosis inhibitor, opposes this function. Hence it is possible that amiloride’s effects on ErbB3 cytoplasmic/membranous accumulation are related to its effects on macropinocytosis. However, ErbB3 was previously shown to endocytose to the cytosol via clathrin-dependent mechanisms while its transportation to the nucleus required binding to importin β [61]. In addition, we cannot rule out a role for uPA in amiloride-induced cell death. Pre-operative plasma levels of uPA and its associated receptor (uPAR) are correlated with PCa disease progression after radical prostatectomy and metastasis [62]. The overall survival rate of PCa patients with elevated serum levels of either uPA or uPAR is significantly lower than that of patients with normal levels [63]. uPA levels are regulated by methylation and transcriptional repression of the uPA promoter in normal prostate whereas in invasive cells, the promoter is unmethylated [64]. uPA also encourages an osteoblastic skeletal response via its growth factor domain, thereby increasing the invasiveness of skeletal and non-skeletal PCa invasiveness [65, 66]. Amiloride inhibits uPA and inactivates uPAR, thereby inducing apoptosis; however, a detailed comparison of amiloride’s effects on NHE1, ENaC or uPA/uPAR is beyond the scope of the current study.

Currently, most stages of PCa are treated with various forms of ADT, including neoadjuvant ADT (NADT). One of the problems with NADT is the likelihood of androgen-independent (AI) clonal formation, resulting in tumor recurrence and CRPC clusters unresponsive to more advanced ADT methods. To lower this possibility, we suggest that using neoadjuvant therapy (NAT) that ‘bypasses’ the androgen receptor may be more beneficial to PCa patients, since it may enable tumors to retain sensitivity to subsequent AR targeting (for example in the case of recurrence after prostatectomy). Our results suggest that the novel combination of lapatinib and amiloride would be suitable for such ‘AR bypass NAT’ for the following reasons: (i) it would reduce tumor size by inducing apoptosis and (ii) should patients develop resistance to it, conventional AR-targeting treatments might still be effective (because lapatinib and amiloride would likely not be used in a post-recurrence setting) (iii) our data shows no effect of the combination on AR.

There may even be some additional effects of this combination. Although lapatinib is usually well tolerated, it has been reported to have cardiotoxic effects in some due to hypokalemia caused by continuous diarrhea. Amiloride, being a potassium sparing diuretic, may prevent this effect by reinforcing hyperkalemia. Moreover, newer and more potent amiloride derivatives such as hexamethylene amiloride (HMA) and 5-(N-ethyl-N-Isopropyl) amiloride (EIPA) exert their anti-cancer effects at nanomolar concentrations and with less toxicity and may also be attractive therapeutic candidates [67, 68]. Both lapatinib and amiloride are oral drugs that can be easily administered. Hence the combination may benefit HSPC patients in a neoadjuvant setting. ErbB3 was previously shown to endocytose to the cytosol via clathrin-dependent mechanisms while its transportation to the nucleus required binding to importin β [61].

## Electronic supplementary material

Below is the link to the electronic supplementary material.

Supplementary Material 1

## Data Availability

All available data has been presented in the manuscript or in the Supplementary Materials. If additional detail is requested, the authors may be contacted for further information. All materials generated for this paper, if available, can be shared with interested individuals against an MTA with the University of California, Davis.

## Abbreviations

PCa: Prostate Cancer
AR: Androgen Receptor
FBS: Fetal Bovine Serum
CSS: Charcoal Stripped Serum
HRG-1ꞵ: Heregulin-One-Beta
EGF: Epidermal Growth Factor
DHT: Dihydrotestosterone
HSPC: Hormone Sensitive Prostate Cancer
CRPC: Castration Resistant Prostate Cancer
PSA: Prostate Specific Antigen
mRNA: messenger RNA
