# Supplementary material for: Amiloride sensitizes prostate cancer cells to the reversible tyrosine kinase inhibitor lapatinib by modulating Erbb3 subcellular localization
Source: Cell Mol Life Sci. 2024 Dec 27;82(1):24. doi: 10.1007/s00018-024-05540-5 (PMC11671466; doi:10.1007/s00018-024-05540-5)
Supplement: Supplementary file 1 — Supplementary Material 1 [file 18_2024_5540_MOESM1_ESM.pdf]

Supplementary Data

SUPPLEMENTARY FIGURE 1. (A) Amiloride did not significantly alter ErbB receptor expression except in one case. RNA from LNCaP, C4-2 and 22Rv1 cells that had been treated for 72h with 75μM amiloride or DMSO was subjected to qPCR to determine amiloride-induced change in ErbB transcript levels. EGFR, HER2, ErbB3 levels were normalized to that of beta-actin. Experiments were repeated in triplicate. Error bars show standard error of the mean. **(B) Differential effect of amiloride in various PCa derived cell lines stimulated with EGF or HRG1.** LNCaP, C4-2 and CWR22-Rv1 cells were treated for 72h with 75μM amiloride or vehicle (DMSO) and stimulated with PBS, EGF or HRG for 15 minutes prior to collection to observe protein levels of ErbB family members. Hsp90 was used as a loading control. Results show that Akt phosphorylation was stimulated by HRG1, but not EGF, both in the presence and absence of amiloride in CRPC lines C4-2 and 22Rv1, whereas in the HSPC line LNCaP, it was activated by HRG1 only in the presence of amiloride, but not under control conditions. In contrast, ERK phosphorylation was stimulated by both EGF and HRG1 in 22Rv1 cells, both in the presence and absence of amiloride, whereas in C4-2 cells, it was only activated by EGF, mainly in amiloride treated cells. On the other hand, in control LNCaP cells, ERK was phosphorylated by EGF only while it was activated by both EGF and HRG1 in the presence of amiloride.

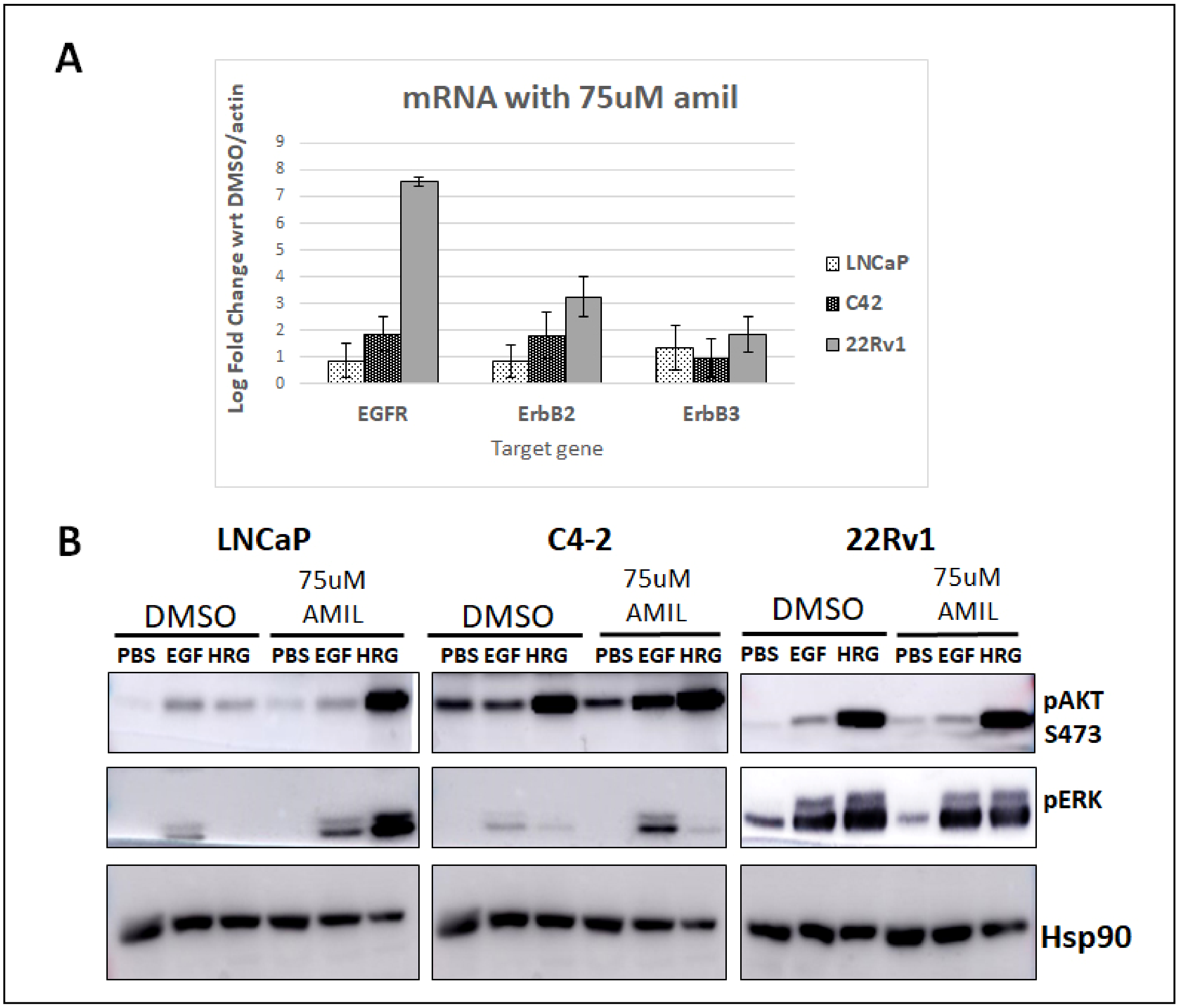

SUPPLEMENTARY FIGURE 2. Co-localization of HER2 and ErbB3 in high dose amiloride. C4-2 cells were treated with DMSO or 75μM amiloride for 72h before being collected, fixed and processed for indirect immunofluorescent microscopy to detect HER2/ErbB3 colocalization using immunofluorescence-specific antibodies as described by us earlier. Results demonstrate that amiloride promoted the co-localization of HER2 and ErbB3 at the plasma membrane. All scale bars = 30μm

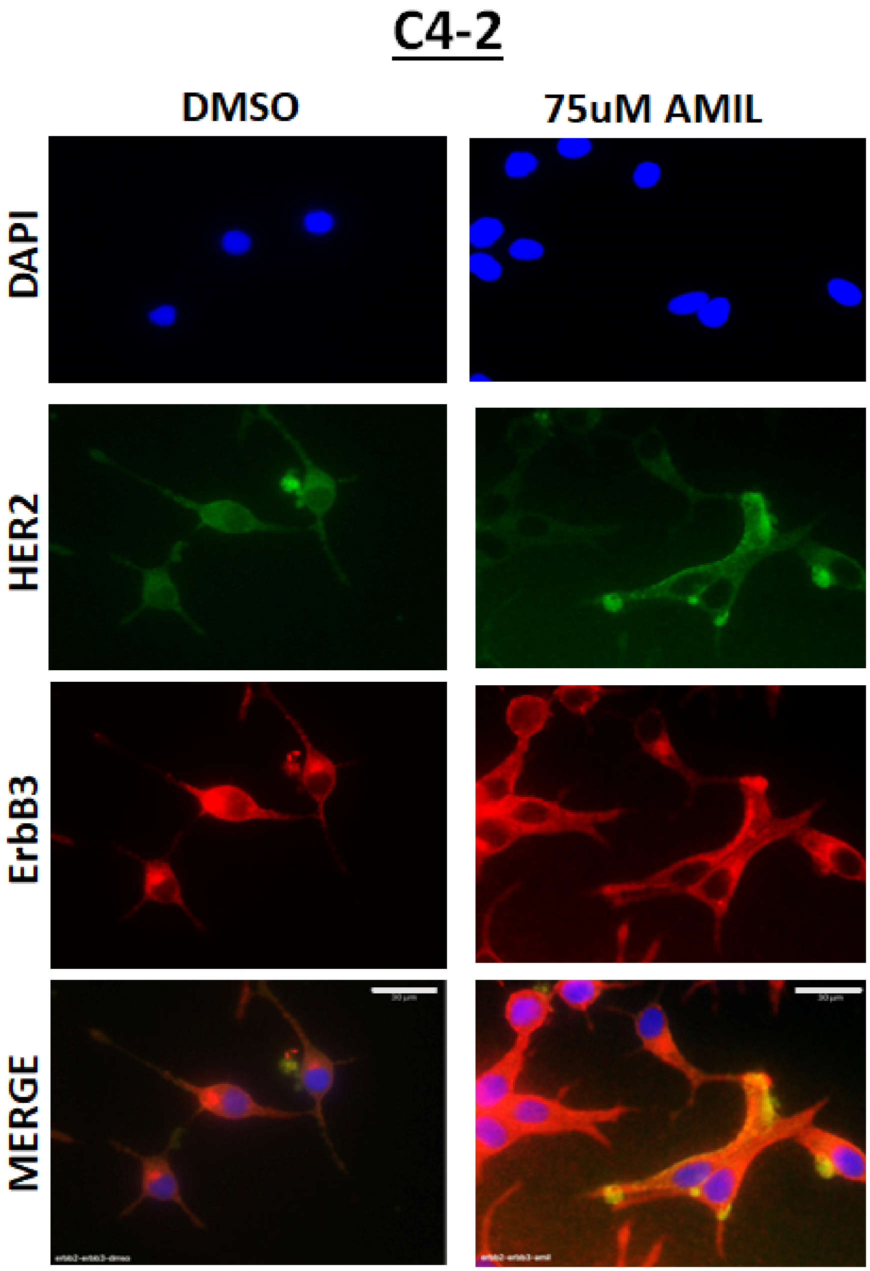

SUPPLEMENTARY FIGURE 3. Low concentrations of amiloride or lapatinib did not affect AR transcriptional activity in 22Rv1 cells. (A) The combination of lapatinib and amiloride increased transcriptional activity on an hPSA promoter in 22Rv1 cells. Readings are the result of experiments performed in triplicate. Cells were transfected with 500ng of the hPSA plasmid for 4h before the media and complexes were aspirated. Cells were allowed to recover overnight in fresh serum-containing media before being treated with 2μM lapatinib or 10μM amiloride as described. Cells were collected 48h after treatment and assayed for luciferase activity using a commercially available kit (Promega) according to manufacturer's instructions. Error bars represent standard deviation. **(B) Amiloride but not lapatinib eliminated nuclear ErbB3 in 22Rv1 cells.** Both the C- and N-terminal ends of ErbB3 are expressed in 22Rv1 cells, indicating that the protein is not truncated in these cells. 22Rv1 cells were plated on coverslips and treated with lapatinib or amiloride for 72h before being fixed and processed for indirect immunofluorescent microscopy (40X) as detailed previously. N- and C-terminal ErbB3 staining (conducted with IF-specific antibodies) appears intensified in cells treated with amiloride compared to lapatinib. **(C) Sigmoidal curves depicting combinatorial effects of 2 μM lapatinib and 10 μM amiloride (low dose) on LNCaP, C4-2 and 22Rv1 cells.** Results from MTT assays of increasing levels of amiloride or lapatinib (shown in the main figures) were compared to that of the combination of 2 μM lapatinib and 10 μM amiloride and showed that the combination had a greater effect on cell viability compared to that of either drug alone.

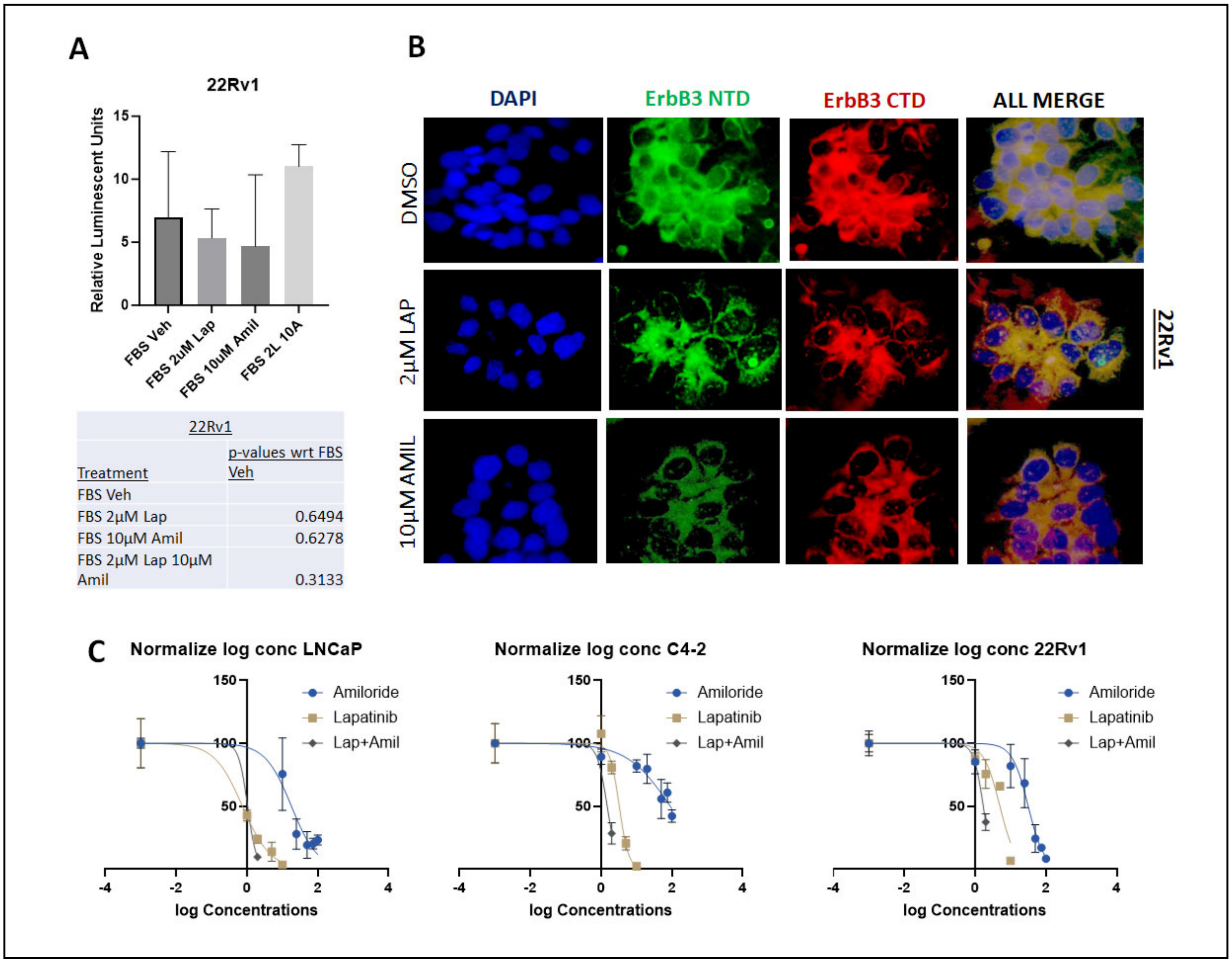

22Rv1	
Treatment	p-values wrt FBS Veh
FBS Veh	
FBS 2μM Lap	0.6494
FBS 10μM Amil	0.6278
FBS 2μM Lap 10μM Amil	0.3133

SUPPLEMENTARY FIGURE 4. Amiloride and lapatinib synergize to increase apoptosis in LNCaP cells. _Note the synergistic increase in apoptosis with the combination_. LNCaP cells were treated with **(A)** 100% DMSO, **(B)** lapatinib, **(C)** amiloride or **(D)** the combination for 72h before being processed for cell death analysis using annexin V (AV) and propidium iodide (PI) staining. The percentage of cells staining for PI or AV were determined by flow cytometry. Q3: unstained cells (live); Q4: Cells staining for AV (early apoptosis); Q2: cells staining for both AV and PI (late apoptosis); Q1: Cells staining for PI only (necrosis). Cells undergoing early or late apoptosis with DMSO treatment was set to 100% and values for the various treatment conditions calculated accordingly. Experiments were performed in triplicate (representative figures shown here).

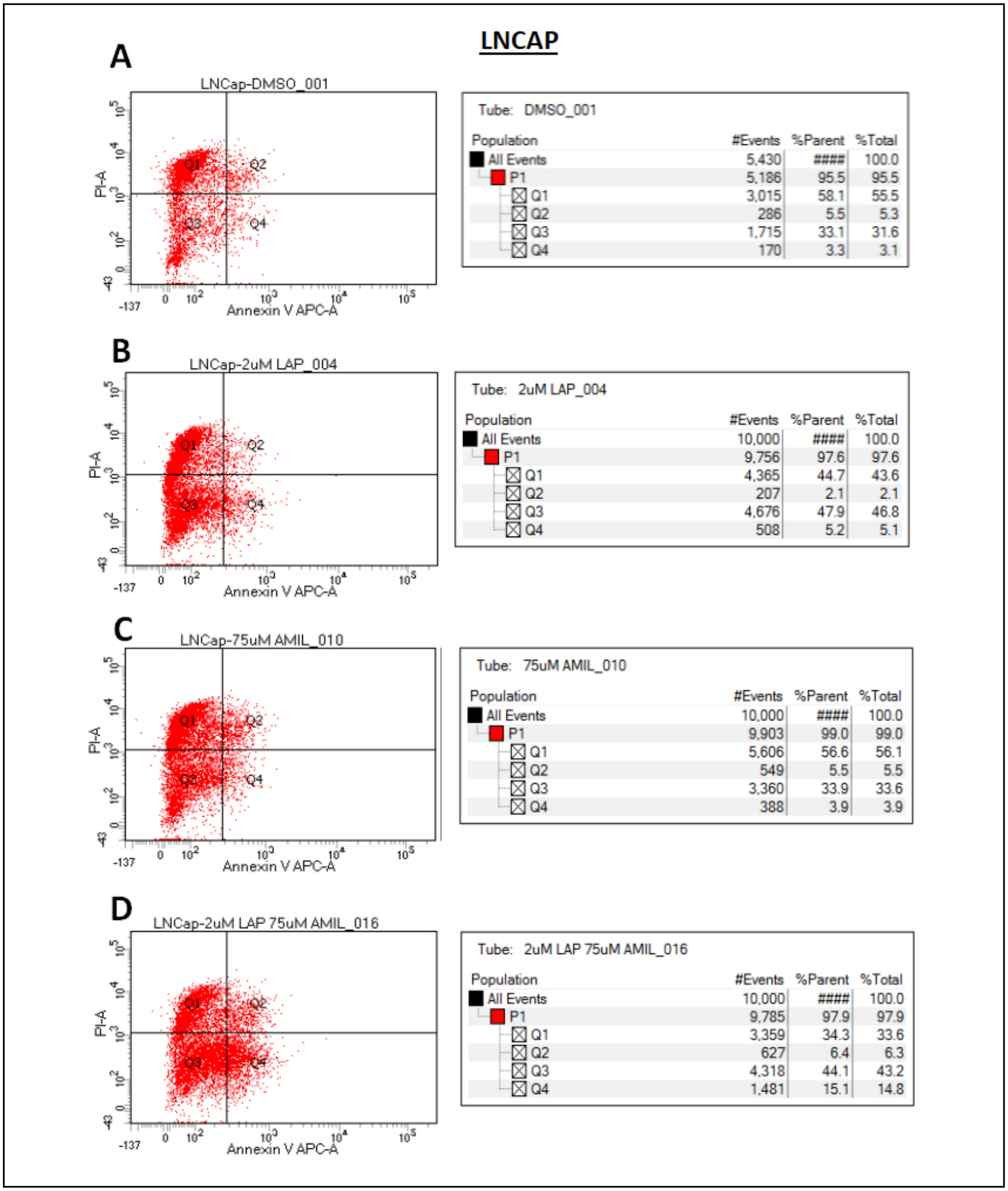

SUPPLEMENTARY FIGURE 5. Lack of synergy in amiloride and lapatinib to increase apoptosis in C4-2 cell line. C4-2 cells (CRPC derivative of LNCaP cells were treated with **(A)** 100% DMSO, **(B)** lapatinib, **(C)** amiloride or **(D)** the combination for 72h before being processed for cell death analysis using annexin V and propidium iodide staining. The percentage of cells undergoing early or late apoptosis with DMSO treatment was set to 100% and values for the various treatment conditions calculated accordingly. Experiments were performed in triplicate. *Unlike LNCaP, C4-2 cells showed no synergistic increase in apoptosis with the combination*.

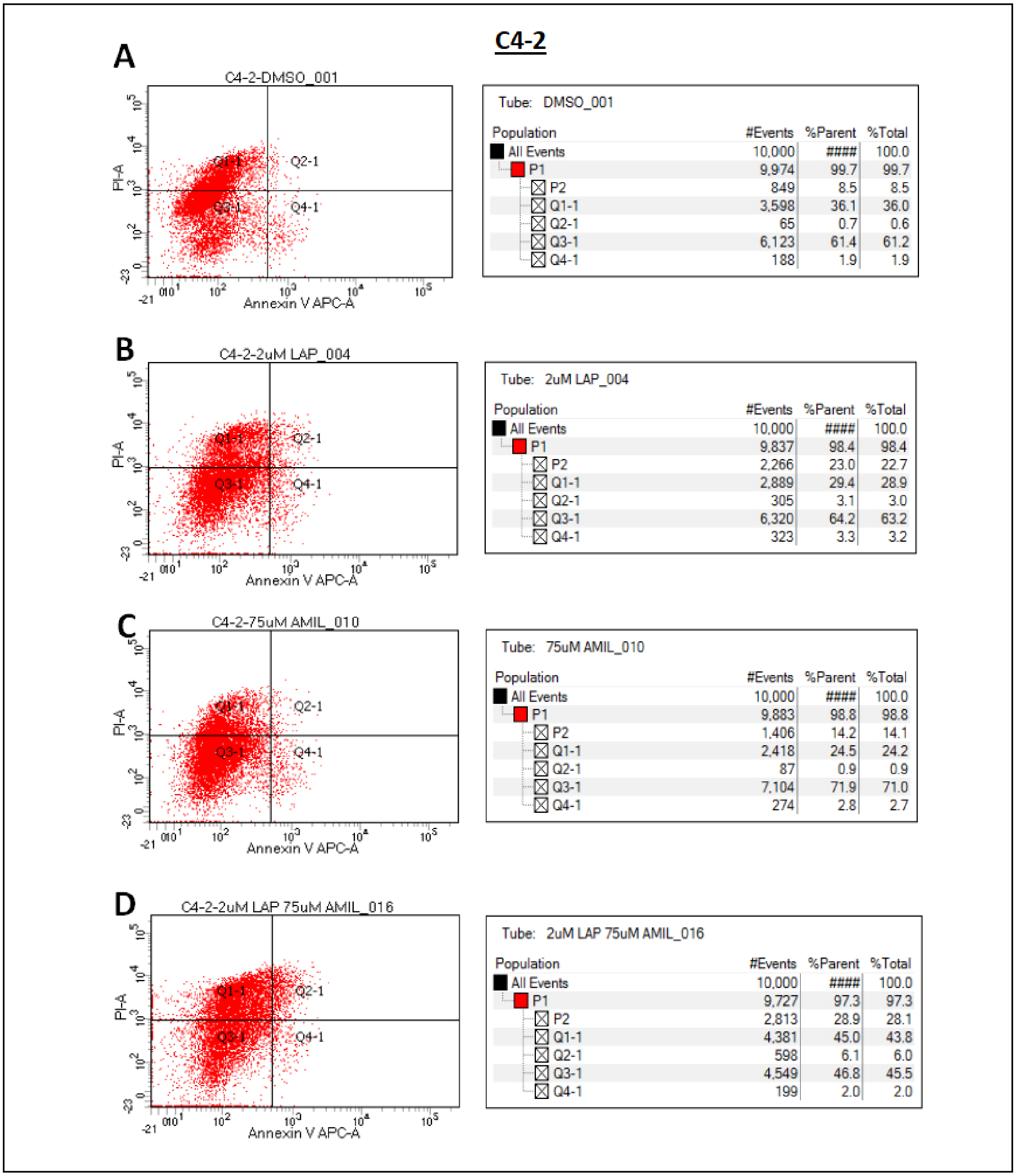

Tube: DMSO_001

Population	#Events	%Parent	%Total
All Events	10,000	####	100.0
P1	9,974	99.7	99.7
P2	849	8.5	8.5
Q1-1	3,598	36.1	36.0
Q2-1	65	0.7	0.6
Q3-1	6,123	61.4	61.2
Q4-1	188	1.9	1.9

Tube: 2uM LAP_004

Population	#Events	%Parent	%Total
All Events	10,000	####	100.0
P1	9,837	98.4	98.4
P2	2,266	23.0	22.7
Q1-1	2,889	29.4	28.9
Q2-1	305	3.1	3.0
Q3-1	6,320	64.2	63.2
Q4-1	323	3.3	3.2

Tube: 75uM AMIL_010

Population	#Events	%Parent	%Total
All Events	10,000	####	100.0
P1	9,883	98.8	98.8
P2	1,406	14.2	14.1
Q1-1	2,418	24.5	24.2
Q2-1	87	0.9	0.9
Q3-1	7,104	71.9	71.0
Q4-1	274	2.8	2.7

Tube: 2uM LAP 75uM AMIL_016

Population	#Events	%Parent	%Total
All Events	10,000	####	100.0
P1	9,727	97.3	97.3
P2	2,813	28.9	28.1
Q1-1	4,381	45.0	43.8
Q2-1	598	6.1	6.0
Q3-1	4,549	46.8	45.5
Q4-1	199	2.0	2.0

SUPPLEMENTARY FIGURE 6. No change in apoptosis in 22Rv1 cells with lapatinib, amiloride or the combination. 22Rv1 cells were treated with **(A)** 100% DMSO, **(B)** lapatinib, **(C)** amiloride or **(D)** the combination for 72h before being processed for cell death analysis using annexin V and propidium iodide staining. The percentage of cells undergoing early or late apoptosis with DMSO treatment was set to 100% and values for the various treatment conditions calculated accordingly. Experiments were performed in triplicate. *The drugs, alone or in combination, did not induce apoptosis in 22Rv1 cells.*

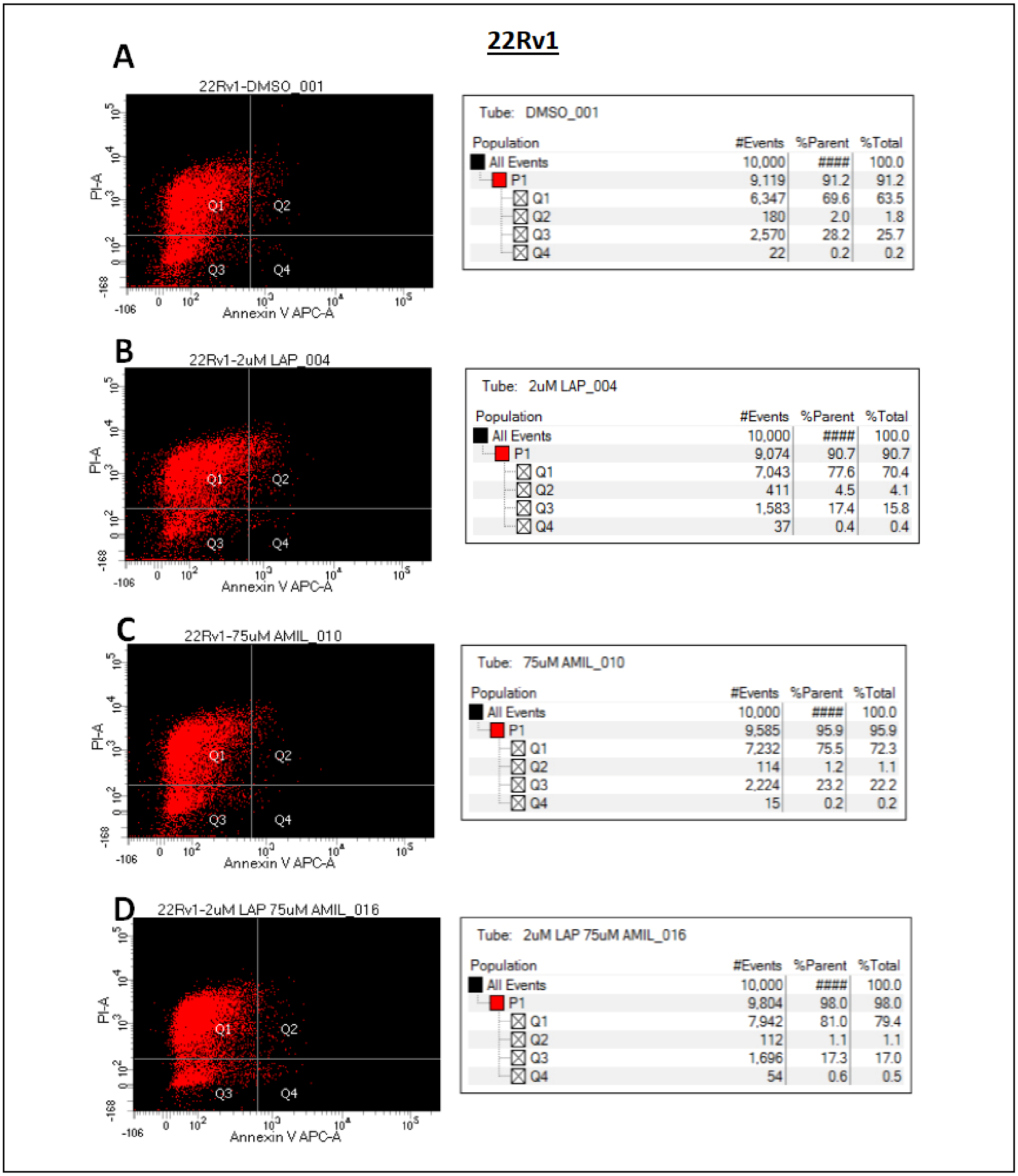

Tube: DMSO_001

Population	#Events	%Parent	%Total
All Events	10,000	####	100.0
P1	9,119	91.2	91.2
Q1	6,347	69.6	63.5
Q2	180	2.0	1.8
Q3	2,570	28.2	25.7
Q4	22	0.2	0.2

Tube: 2uM LAP_004

Population	#Events	%Parent	%Total
All Events	10,000	####	100.0
P1	9,074	90.7	90.7
Q1	7,043	77.6	70.4
Q2	411	4.5	4.1
Q3	1,583	17.4	15.8
Q4	37	0.4	0.4

Tube: 75uM AMIL_010

Population	#Events	%Parent	%Total
All Events	10,000	####	100.0
P1	9,585	95.9	95.9
Q1	7,232	75.5	72.3
Q2	114	1.2	1.1
Q3	2,224	23.2	22.2
Q4	15	0.2	0.2

Tube: 2uM LAP 75uM AMIL_016

Population	#Events	%Parent	%Total
All Events	10,000	####	100.0
P1	9,804	98.0	98.0
Q1	7,942	81.0	79.4
Q2	112	1.1	1.1
Q3	1,696	17.3	17.0
Q4	54	0.6	0.5
